# Microbial elicitors to metabolic reprogramming: an integrative model of plant-microbe interactions

**DOI:** 10.3389/fmicb.2026.1816468

**Published:** 2026-04-28

**Authors:** Dulali Bala Hembram, Soumya Prasad Panda, Basanta Kumar Das, Dhananjay Soren, Nihar Ranjan Singh

**Affiliations:** 1 Department of Botany, Ravenshaw University, Cuttack, India; 2 Department of Botany, Shailabala Women's Autonomous College, Cuttack, India; 3 Centre of Excellence in Environment and Public Health, Ravenshaw University, Cuttack, India; 4 Aquatic Environmental Biotechnology Division, ICAR-Central Inland Fisheries Research Institute, Kolkata, West Bengal, India; 5 Enzymology and Cancer Biology Laboratory, Department of Zoology, Ravenshaw University, Cuttack, India

**Keywords:** elicitors, key genes, microbial consortium, secondary metabolite, system biology, transcription factors

## Abstract

Plant growth, soil health, and crop productivity with nutritional quality can be significantly enhanced by employing microbial consortia that incorporate diverse microorganisms with complementary functions. Plants produce various types of secondary metabolites such as terpenoids, alkaloids, phenolics, essential oils, and other metabolites through various cellular mechanisms, which are often stimulated by microbial interactions. These metabolites exert beneficial effects on plants and perform multiple roles in agriculture, contributing significantly to growth and economy. This review summarizes microbial consortia-mediated enhancement of plant health and their intricate interactions with host plants. Beneficial microbes of a consortium trigger complex signaling cascades leading to a dynamic regulatory strategy through which plants enhance their secondary metabolite synthesis. Secondary messengers and hormonal cross-talk further integrate the signal to transcription factors, which play a central role in activating or repressing the key genes of the metabolic pathways. Thus, the interplay of microbial signal, secondary messengers, hormonal cross-talk, and key metabolite genes forms the basis of plant secondary metabolite biosynthesis. In addition, recent advances in systems microbiology, including metagenomics, metatranscriptomics, and metabolomics, have enabled a holistic understanding of microbial community dynamics and their collective role in regulating secondary metabolism.

## 1 Introduction

Soil is one of the important constituents of the environment, supporting a diverse array of life forms, including plants and microorganisms. Soil microbes play an essential role in shaping soil biomes thereby sustaining energy flows that support entire ecosystems. By facilitating nutrient availability through mineralization and by degrading pesticides, insecticides and other toxic compounds, they contribute significantly to improved plant productivity (Chowdhury et al., 2008) Therefore, microbial activity is indispensable for soil ecosystem as well as plant growth (Comite et al., 2021; Silletti et al., 2021).

The normal microflora of soil represents a complex community comprising thousands of microbial species with numerous potential interactions among them. These interactions play a fundamental role in determining the properties of the soil microbiome. Plant growth promoting microorganisms (PGPM) are essential constituents of soil microbiota due to their ability to enhance root surface area, plant growth and productivity, nutritional quality along with resistance to pathogen attack and abiotic stress. They comprise a diverse array of microorganisms such as Arbuscular Mycorrhizal fungi (AM fungi), nitrogen-fixing bacteria, phosphate-solubilizing microorganisms, plant growth-promoting rhizobacteria, actinomycetes, biocontrol strains, and endophytic bacteria (Vejan et al., 2016).

From a systems microbiology perspective, these microbial communities function as highly interconnected networks where community-level interactions, rather than individual species, govern ecosystem processes. Understanding these emergent properties requires integration of multi-omics datasets to decipher microbial co-occurrence patterns, functional redundancy, and metabolic interdependencies within microbial consortia.

This review emphasizes that the collective functions of multiple microbial species, when present in the form a consortium play a crucial role in enhancing plant growth and regulating plant metabolic processes.

## 2 Microbial consortium

A microbial consortium is a group of compatible strains with different functional properties that collaborate and contribute as a community to achieve collective goals. Natural consortia consisting of bacteria and fungi are commonly found in various environments, including both soils and plants (Zhou et al., 2018, 2022; Ayala-Campos et al., 2022). The coexistence of multiple species within a consortium having synergistic interactions allows them to occupy a broader range of ecological niches, facilitating numerous beneficial functions.

Besides, they are also reported to exhibit high degree of self-regulatory system that enhance resource utilization through efficient metabolite exchange. They show remarkable adaptability and viability with changes in environmental factors such as pH, temperature, moisture, and organic compounds. Their potential applications are vast including ecological restoration and rejuvenation, bioremediation of soil contaminants, soil restoration, waste and sewage treatment, climate change mitigation and over all environmental monitoring. A consortium can be classified in to three categories: artificial, synthetic and semi-synthetic. Artificial consortia consist of specific microbial strains designed to perform specific functions that rarely coexist under natural habitats. Synthetic consortia are composed of genetically modified microorganisms, while semi-synthetic consortia include both natural and genetically modified microorganisms (Bernstein and Carlson, 2012). The application of a single microbial strain often fails to colonize the plant rhizosphere and does not safeguard plants against a wide range of pathogen attacks. So, the development of an effective consortium has emerged as an important strategy for enhanced resilience against pathogen threats and to function as a dynamic biocontrol agent (Rivett et al., 2018; Yaduwanshi et al., 2021; Singh et al., 2025).

### 2.1 Promotive functions of microbial consortia

Arbuscular Mycorrhizal Fungi (AMF) and plant growth promoting bacteria are the most pertinent group of microorganisms inhabiting the soil. *Trichoderma* sp. a prevalent soil fungus is commercially produced and widely used in agriculture for the control of plant diseases (Monte and Hermosa, 2021). It works by establishing various interactions such as parasitism, competition or antagonism and induces resistance in plant by stimulating the bio-synthesis of enzymes, peptides, proteins, volatile organic compounds and secondary metabolites (Woo and Pepe, 2018).

When a microbial consortium consisting of three bacteria *Enterobacter* sp., *Microbacterium arborescens*, and *Serratia marcescens* was introduced in to wheat field, it led to remarkable enhancement in plant growth, productivity and nutrient uptake process (Kumar et al., 2017).

Similarly, a consortium of *Mesorhizobacterium* sp., *Pseudomonas* sp., *Burkholderia* sp., and AMF increased growth and productivity in chick pea plant under rainfed conditions (Laranjeira et al., 2021). Applications of microbial consortia as a coating of fertilizers in potato crops changed the richness and composition of soil microbes particularly prokaryotes and was directly associated to the potato productivity (Overbeek et al., 2021).

A soil bacterium *Bradyrhizobium* sp. LSBR-3, in combination with *Pseudomonas oryzihabitans* LSE-3 significantly enhanced various traits in soybean plants including growth and nutrient accumulation compared to single strain inoculation. This consortium resulted in an approximately 11% increase in plant yield compared to control plots (Kumawat et al., 2022). Likewise, consortium of four mycorhhizal fungi (*Funneliformsis constrictum, F. mosseae, Gigaspora margarita*, and *Rhizophagus irregularis*) along with the bacterium *Beauveria bassiana* promoted the growth of cotton plants which increased the protein and carbohydrates contents and reduced the growth of *Spodoptera littoralis* (Metwally et al., 2022). In a related study, four species of *Beauveria* combined as a consortium showed 100% mortality of moth *Plutella xylostella*, demonstrating the effect of synergistic interaction (Soth et al., 2022). Thus, a consortium harnesses the potential of multidisciplinary synergistic microorganisms for preserving the soil health and its microbiota (Santoyo et al., 2021; Woo et al., 2023; Hegde and Monisha, 2024).

Among the diverse microbial groups, plant growth promoting rhizobacteria represent one of the most significant functional categories because of their close association with plant roots and role as modulators in plants metabolic pathways.

## 3 Plant growth promoting rhizobacteria as microbial consortia

Plant growth promoting rhizobacteria (PGPR) are among the most prominent agronomically useful soil microbes facilitating plant growth by colonizing plant roots. They are highly significant and have been increasingly used in agriculture as they establish mutualistic interactions with plants encouraging root and shoot growth and for their vital ability to counteract abiotic stress (Gul et al., 2023) leading to replacement of chemical fertilizers (Vishwakarma et al., 2020; Elnahal et al., 2022).

PGPR are primarily represented by species from genera such as *Bacillus* sp., *Pseudomonas* sp., *Lactobacillus* sp., *Azospirillum* sp., *Acetobacter* sp., *Arthrobacter* sp., *Ochrobactrum* sp., *Beijerinckia* sp., *Azoarcus* sp., *Stenotrophomonas* sp., *Paenibacillus* sp., *Rhodococcus* sp., *Derxia* sp., *Burkholderia* sp., *Acinetobacter* sp., *Klebsiella* sp., *Enterobacter* sp., *Pantoae* sp., *Gluconacetobacter* sp.*, Alcaligens* sp., *Herbaspirillum* sp., *Agrobacterium* sp., *Serratia* sp., and *Rhizobacterium* sp. (Vega-Celedon et al., 2021).

PGPR are categorized as (a) symbiotic bacteria which live inside plants and exchange metabolites with them and (b) free-living rhizobacteria, those reside outside plant cells. They promote plant growth through various ways, which can be summarized based on their mechanisms of function, like (i) synthesis of growth promoting substances, (ii) nutrient cycling, (iii) induction of plant immunity, (iv) prevention of plant diseases, and (v) nitrogen fixation.

Symbiotic and endophytic bacteria inhabiting the rhizosphere produce phytohormones that facilitate nutrient absorption, expansion of root and shoot system, seed germination, flowering and overall plant growth. Similarly, plant promotes PGPR growth by releasing root exudates containing various substances such as carbohydrates, organic acids, minerals and other metabolites that serve as nutritional resources for microorganisms (Siddiqui et al., 2021).

### 3.1 Mechanisms of action of plant growth promotion

**Figure 1:**
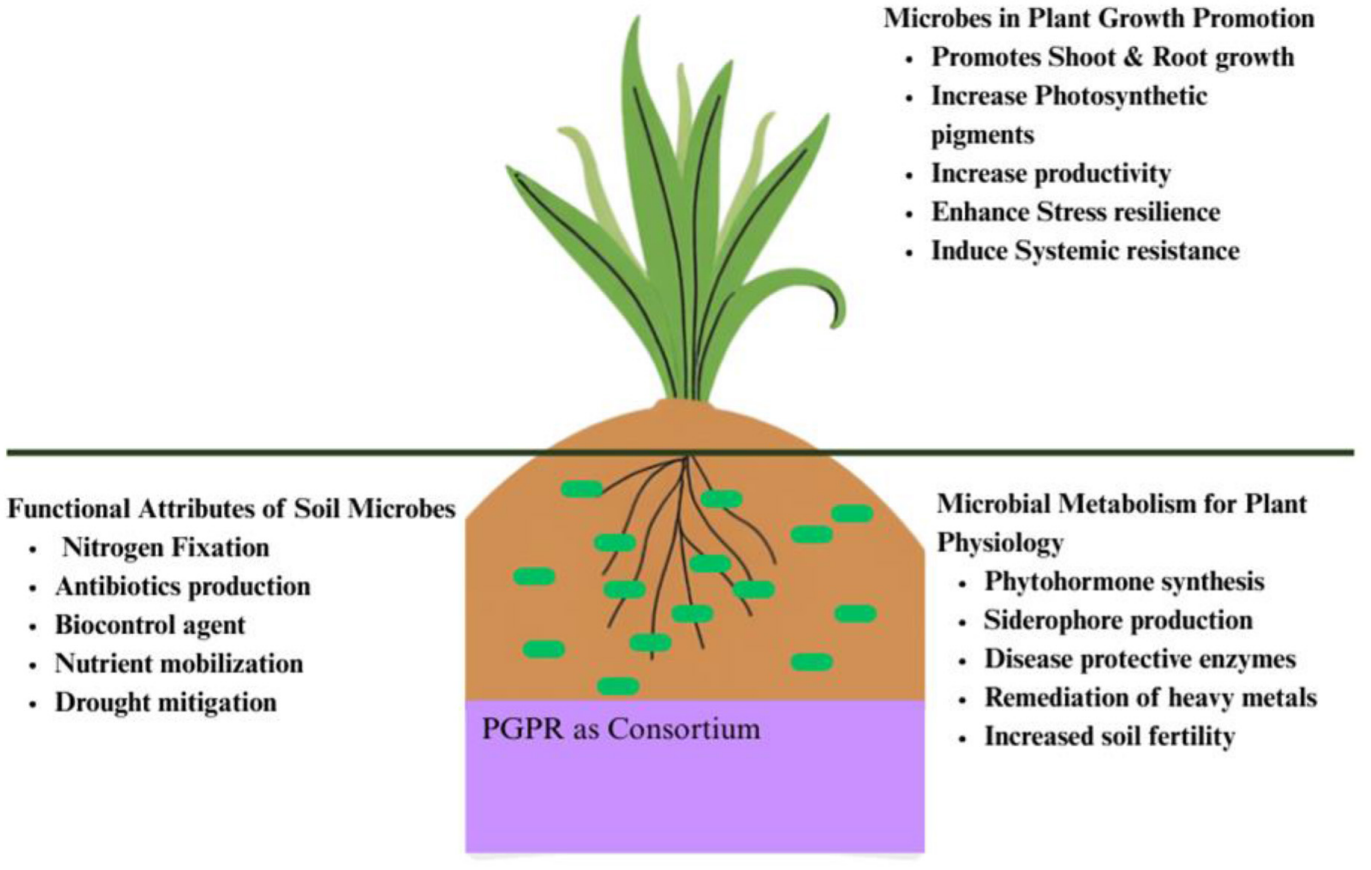
PGPR as microbial consortium. PGPR-plant interactions in the rhizosphere drive a cascade of beneficial events including nutrient solubilization, phytohormone regulation, activates defense mechanisms ultimately boosting plant growth and resilience.

PGPR influence plant growth through direct and indirect stimulation once they initiate interaction with plants. Direct mechanisms promote plant growth by providing solubilized minerals that stimulate aerial biomass production in shoot and enhance root development through biosynthesis of phytohormones such as gibberellins, auxins, indole-3-acetic acid (IAA), cytokinin, abscisic acid, and ethylene. *Acinetobacter* sp., *Pseudomonas* sp., *Rhizobium* sp., *Azospirillum* sp., *Bacillus* sp., and *Klebsiella* sp. are the most common genera linked to the biosynthesis of IAA in the rhizosphere of different crops and vegetables (Choudhary et al., 2018). Auxin is the key molecule that regulates most plant processes directly or indirectly. Several bacteria secrete auxins that act as signaling molecules to facilitate communication among bacteria for coordinated functions. When plants encounter stressed conditions, they produce high levels of ethylene which inhibits the root elongation or nitrogen fixation in legumes contributing to early senescence. PGPR produces enzyme 1-aminocyclopropane 1-carboxylate (ACC) deaminase, thus reducing ethylene levels that providentially support the root development (Singh and Jha, 2017). Direct mechanism improves growth efficiency by releasing or solubilizing phosphates and other useful nutrients such as potassium, zinc and silicon, chelating metal ions and other micronutrients such as boron, calcium, magnesium, and copper thereby increasing the availability of oxygen (Rehman et al., 2018; Sidhu et al., 2025; Figure 1).

Indirect mechanisms alter the diversity, composition and functioning of rhizosphere environment and boost the host's inherent resilience in several ways. PGPR secrete hydrolytic enzymes (chitinases, cellulases) and release substances like siderophores, antibiotics, pigments, organic acids (malic, citric, lactic, formic, gluconic, oxalic, and 2-keto gluconic), water-soluble vitamins (niacin, thiamine, and biotin) or antioxidants and volatile organic compounds such as monoterpene alcohols (Chandran et al., 2021). Organic acid lowers the pH of rhizosphere soil thereby dissolving insoluble inorganic phosphates and make them available to plants (Ríos-Ruiz et al., 2025). The PGPR activate release of these substances as protective mechanisms to mitigate the abiotic stress factors such as drought or aridity which is caused by dryness, high salt concentration, and elevated environmental temperature (Ríos-Ruiz et al., 2025).

Various pathogens including bacteria, viruses, fungi, nematodes, protists, insects, and viroids can cause biotic stress, ultimately reducing crop productivity (Haggag et al., 2015). To counter the adverse conditions, microorganisms such as *Bacillus* sp., produce volatile organic compounds like 2-butanediol and acetoin, preventing the spread of pathogens and aid in plant development (Santoro et al., 2016).

Most of the *Pseudomonas* species produce antibiotics such as Oomycin A, Cepaciamide A, Ecomycins, and Viscosin with other chemical compounds including Pyrrolnitrin, Pyoluteorin, 2,4-diacetylphloroglucinol, and Rhamnolipids. Additionally, *Bacillus* sp. produces a number of lipopeptide antibiotics such as Surfactin and Bacillomycin along with other antifungal agents indicating their role as excellent biocontrol agents to suppress pathogen mediated infections (Liu et al., 2022a; Rai et al., 2023; Xiang et al., 2023). PGPR also secrete nitrilase enzyme that helps in removing nitrile content from the cropland (Vaishnav et al., 2022). Although both direct and indirect mechanisms significantly contribute to plant health, nitrogen fixation remains a crucial process performed by all PGPR (Fouzia et al., 2015).

Due to their versatile roles, PGPR are highly essential in agronomy. They facilitate the circulation of energy and resources in an ecosystem by establishing a network with various microbial communities (Sylia et al., 2022). Employing PGPR as a microbial consortium, therefore, appears to be a promising approach to enhance plant growth and resilience (Table 1).

**Table 1:** PGPR strain promoting plant growth both by direct and indirect mechanisms.

PGPR strain(s)	Host plant species	Growth-promoting substances/characteristics	References
*Acinetobacter* sp.	Alfaalfa (*Medicago sativa* L.)	IAA, siderophore production, phosphate solubilization	(Tafaroji et al., 2022)
*Azotobacter chroococcum*	Canola (*Brassica napus* L.)	Gibberellin, IAA, kinetin, antioxidant activity	(Abdel Latef et al., 2021)
*Azotobacter vinelandii*	Maize (*Zea mays* L.)	IAA, phosphate solubilisation, nitrogenase activity	(Jain et al., 2021)
*Azospirillum amazoense*	Rice (*Oryza sativa* L.)	IAA and nitrogenase activity	(dos Santos Ferreira et al., 2022)
*Bradyrhizobium* sp.	Chickpea (*Cicer arietinum* L.)	IAA, siderophore production, HCN, ammonia, and exopolysaccharide	(Kiruthika and Arunkumar, 2021)
*Bradyrhizobium* sp. 750	Rice (*Oryza sativa* L.)	Heavy metals	(Fagorzi et al., 2018)
*Bacillus subtilis*	Soybean (*Glycine max*)	Ion homeostasis, antioxidant and antifungal activity, osmoregulation and methylglyoxal detoxification	(Hasanuzzaman et al., 2022)
*Azotobacter* sp. (SR-4)	Calabash (*Lagenaria siceraria*) and Okra (*Abelmoschus esculentus*)	Nitrogenase activity	(Pandove et al., 2023)
*Azotobacter salinestris*	Tomato (*Solanum lycopersicum* L.)	IAA, ammonia, ACC deaminase, and exopolysaccharides	(Danish et al., 2022)
*Bacillus subtilis* DR2	Coriander (*Coriandrum sativum*)	Siderophore production	(Singh et al., 2022b)
*Burkholderia* sp.	Tea (*Camella sinensis*)	IAA, phosphate and heavy metal solubilization, ACC deaminase, siderophore	(Han et al., 2021)
*Brevibacillus* sp. Desert YSK *Rhizobium* MAP7	*Lactuca sativa* L.	Zinc resistance and IAA production	(Mowafy et al., 2023)
*Bravibacterium* sp.	Scots pine (*Pinus sylvestris*)	Siderophore production	(Lutfullin et al., 2022)
*Bradyrhizobium japonicum* *Bacillus megaterium*	*Glycine max* L.	IAA, siderophore production, phosphate solubilization	(Miljaković et al., 2022)
*Azospirillum* sp.	Strawberry (*Fragaria x ananassa*)	Siderophore production	(Sivasakthivelan et al., 2023)
*Bradyrhizobium japonicum*	Mung bean (*Vigna radiata*)	IAA	(Zveushe et al., 2023)
*Bacillus subtilis* SL-44	Maize (*Zea mays* L.)	IAA, siderophore, phosphate solubilization	(Xiang et al., 2023)
*Variovorax paradoxus*	Cucumber (*Cucumis sativus*)	IAA, siderophore production	(Flores-Duarte et al., 2022)
*Serratia marcescens*	Sunflower (*Helianthus annus*) and Maize (*Zea mays*)	IAA, siderophore, and HCN	(Sriwati et al., 2023)
*Stenotrophomonas* sp. CV83	Rice (*Oryza sativa* L.)	IAA, ACC deaminase, and siderophore production	(Sharma et al., 2023)
*Rhizobium leguminosarum* Thy2	Wheat (*Triticum aestivum*)	Siderophore production, nitrogen fixation ability to mitigate Cd stress	(Nofal and Attia, 2023)
*Rhizobium* E20-8	Maize (*Zea mays* L.)	IAA, antioxidant activity, and siderophores	(Cruz et al., 2023)
*Rhizobium* sp.	Lentil (*Lens culinaris* Medik L.)	IAA, HCN, exopolysaccharides, ammonia, and siderophores	(Banjare et al., 2023)
*Pseudomonas* sp. K32	Rice (*Oryza sativa* L.)	Pb and Cd stress	(Rai et al., 2023)
*Pseudomonas aeruginosa*	*Lilium davidii*	IAA, antifungal activity, ACC deaminase, siderophores production, and phosphate solubilization	(Khan et al., 2022)
*Flavobacterium* sp.	Common beans (*Phaseolus vulgaris* L.)	IAA and siderophores	(Feng et al., 2023)
*Klebsiella oxytoca*	Maize (*Zea mays* L.)	IAA, phosphate solubilization, and nitrogenase activity	(Khalifa and Aldayel, 2022)
*Mesorhizobium* sp.	Chick pea (*Cicer arietinum* L.)	IAA, siderophores, ammonia, exopolysaccharides, and HCN	(Yadav et al., 2022)
*Enterobacter asburiae*	Chick pea (*Cicer arietinum* L.)	IAA, siderophores, phosphate solubilization, ammonia, HCN, and exopolysaccharides	(Saikia et al., 2023)
*Bacillus pumilus*	Rapeseed (*Brassica napus*)	IAA, HCN production, phosphate solubilization, antifungal activity	(Lipková et al., 2021)

### 3.2 Multi-omics approaches in microbial consortia and network regulation

Recent advances in high-throughput omics technologies have significantly enhanced our understanding of microbial consortia functioning. Metagenomics provides insights into microbial diversity and genetic potential, while metatranscriptomics reveals actively expressed genes under specific environmental conditions. Additionally, metaproteomics and metabolomics reveal functional proteins and metabolic fluxes that govern microbial interactions (Ramzan et al., 2026).

Plant secondary metabolites synthesized by microbial consortia can be investigated through metabolomic analysis which involves both data acquisition and subsequent processing. Data processing typically comprises steps such as data normalization, peak alignment, and data scaling (Tahir et al., 2019). A range of specialized software tools including MZmine2, XCMS, Open MS, MS-DIAL, and Decon2Ls are available to facilitate these processes and can handle datasets generated from both NMR and MS.

Detailed dynamics between plant secondary metabolites and consortia are of considerable interest for understanding plant-microbe interactions. Importantly, tracing PSMs at the single cell level would enable *in-situ* observation of metabolic processes and facilitate precise tracking of metabolites during these interactions. Therefore, high resolution detection systems combined with innovative cell sampling technique are essential for profiling and tracing the metabolite at the single cell scale. Since PSMs are the result of both the host plants and combined influence of the consortium, effective frameworks are required to ensure accurate metabolites tracing and elucidation of their biological function (Pang et al., 2021).

The Tracking Root Interaction System (TRIS; Massalha et al., 2017) and the Root-Microbe Interaction Chip (RMI-Chip; Noirot-Gros et al., 2020) are biosensors that facilitate real time investigation of root microbiome interactions. These platforms provide direct imaging and enable spatiotemporal, non-destructive, *in situ* examination of samples. Furthermore, whole sample metabolic profiling of non-sterile rhizosphere soil have been investigated as an additional approach (Pétriacq et al., 2017). Collectively, these novel methodologies allow researchers to identify which substrates microbes prefer and reveal the interactions between PSMs and microbiome.

Apart from all these existing approaches, there is also a pressing need of software platforms capable of integrating the information of both microbes and PSMs. Statistical methods for data integration have been developed to identify the potential molecular markers driving these interactions (Lamichhane et al., 2018). However, there remains a significant gap in our understanding of how to effectively integrate multi omics datasets such as those derived from proteomics and transcriptomics. Several metabolite profiling methods are available for instance, ESI-triple quadrupole-linear ion trap Q-MS (Luo et al., 2016) and ESI-QqTOF-MS (Chen et al., 2013) which enable large scale detection, quantification and identification of common metabolites. Yet, no comprehensive database currently exists that links plant-microbes consortium data with PSM information under varying environmental conditions. Such a resource is urgently needed to facilitate large scale multi-omics integration in plant microbiome research.

Transcriptome sequencing (RNA-seq) enables the study of not only transcription factors, functional genes and differentially expressed genes but also provides valuable insights in to secondary metabolic pathways. Owing to its broad applicability, RNA-seq has become widely adopted discipline in plant molecular biology. Gene expression in plants exhibits temporal and spatial variation across different tissues and organs making transcriptomic analysis essential for understanding developmental and physiological processes. Nevertheless, elucidating biosynthetic pathways in plants remains challenging due to the functional diversity and genetic complexity of these pathways compared with other biological systems (Singh et al., 2022c).

Transcriptional data are strongly influenced by factors such as developmental stage, type of tissue and physiological conditions underscoring the importance of careful sample collection and analysis. Next Generation Sequencing (NGS) technologies generate short reads, which restricts analysis to local gene structures and prevent full length transcript resolution. This limitation has driven development of third-generation sequencing platforms that produce long reads. Technologies such as Oxford Nanopore Sequencing and PacBio single-molecule real time sequencing offer significant advantages for transcriptomics though their wide spread use is constrained by high costs (Guo et al., 2021).

More recently, single cell RNA-seq has gained popularity as a powerful approach to study transcriptomes at the resolution of individual cells (Shaw et al., 2021). In addition, *de novo* transcriptomic studies in medicinal plants of Solanaceae have been reported focusing on secondary metabolite biosynthetic pathways through RNA sequencing, transcriptome assembly and functional annotation of transcriptomic data. These advances highlight both the immense potential and challenges associated with applying multi omics system to unravel complex plant metabolic networks (Dasanya and Hewadikaram, 2024).

## 4 Plant secondary metabolites

Plant bioactive compounds are categorized into two major groups as primary and secondary metabolites. Plant secondary metabolites (PSMs) are metabolic intermediates or byproducts that are non-essential for growth and survival but play crucial roles in interactions with other microorganisms and perform specific functions under certain conditions (Yang et al., 2018; Ahanger et al., 2020). These species-specific metabolites mediate multiple roles in agriculture, contributing significantly to plant growth. They are primarily involved in defense response signaling, interactions between plants and microbes, abiotic stress tolerance, minerals transport, facilitating fertilization or seed dispersal, and also contribute to plant coloration, fragrances, and flavors (Chen et al., 2022).

Plants produce various types of secondary metabolites. Based on their chemical structures, plant secondary metabolites are classified into three major classes: terpenoids, phenolic compounds and nitrogen-containing compounds (Hussein and El-Anssary, 2019). The accumulation process in plants is influenced by both inherent internal factors and external physical parameters. Internal factors include plant genotype, age, stage of development and the specific plant organs. External factors are the physical parameters which include intensity of sun light, temperature, soil texture, water holding capacity, conductance, minerals as micronutrients, salt concentration, pH and the presence of living microorganisms (Yang et al., 2018; Li et al., 2021). These metabolites are highly reactive and their accumulation can lead to changes in physiological and morphological characteristics of plants such as difference in number and surface area of leaf, plant height and productivity (Jeyasri et al., 2023). Various plant secondary metabolites (PSMs) perform distinct functions. For instance, phenolic compounds enhance plant's resistance to abiotic stress such as extreme dryness, salinity and heavy metals exposure (Mekureyaw et al., 2022). Similarly, alkaloids protect plants from insects and pest diseases (Zeng et al., 2013; Nalini et al., 2025).

Several studies have reported that microorganisms play a crucial role in the accumulation and regulation of plant secondary metabolites (Song et al., 2023; Toppo et al., 2023; Bankar and Chapadgaonkar, 2025; Rana et al., 2025). Among the microorganisms reported, rhizospheric and endophytic microorganisms play important role. It is reported that amount of secondary metabolites (polyphenols, flavonoids, triterpenoids, and lignans) synthesis in plant of the Schisandraceae family was found to be increased in presence of endophytic microbes *Hebeloma* and *Clavulinopsis* sp. (Qin et al., 2024).

Endophytes colonize plant organs without causing any obvious plant diseases and produce a variety of metabolites such as plant growth regulators, antibiotics, alkaloids and enzymes (Shao et al., 2023). On the other hand, rhizospheric microorganisms increase plant's tolerance to biotic and abiotic stress and simultaneously facilitating nutrient absorption and enhancing plant yield and quality.

Understanding the regulatory mechanisms of microbes-mediated secondary metabolite synthesis in plants is immensely essential since these compounds have significant pharmacological significance. This review elaborates primarily on the (i) roles of microorganisms in the accumulation of these metabolites and (ii) regulatory mechanisms of microbes induced biosynthesis in plants.

### 4.1 Role of microorganisms in shaping plant secondary metabolites

Microorganisms associated with plants have significant influential role on synthesis of plant secondary metabolite, altering their physiological process in different natural habitats. The major classes of secondary metabolites in plants are terpenoids, phenolic compounds, alkaloids and essential oils.

#### 4.1.1 Terpenoids

Terpenoids are among the most abundant plant products, exhibiting vast structural diversity and widespread distribution in plant tissues. They play multifaceted roles in plant metabolism and have significant applications in sustainable agriculture. Terpenoids regulate plant growth by influencing hormone synthesis and provide allelopathic protection against herbivores and pathogenic microbes (Umehara et al., 2008). Terpenoid biosynthesis in plants occurs via the mevalonate (MVA) pathway in the cytoplasm or the methylerythritol 4-phosphate (MEP) pathway in plastids.

On exposure to drought stress, secondary metabolites like fatty acids and diterpenoids are synthesized and accumulation of these compounds facilitate recruitment and abundance of *Proteobacteria* sp. and *Acidobacteria* sp. (Thomas-Barry et al., 2021). Correspondingly, bacteria such as *Acinetobacter pittii, Bacillus pumilus*, and *Pseudomonas fluorescens* have been reported to produce terpenoids or their precursor molecules in some medicinal plants (Xie et al., 2019b; Tripathi et al., 2020). Similarly, *Pseudomonas fluorescens* strain ALEB7B, an endophyte has been reported to enhance the production of sesquiterpenoids in a Chinese medicinal plant (Zhou et al., 2018).

Inoculation with *Mucilaginibacter* sp. and *Pseudomonas* sp. at the flowering stage in *Cannabis sativa* resulted in an increase in terpenes by approximately 23% and 18% over the control, respectively. Out of all the terpene profiles studied, inoculation with *Mucilaginibacter* sp. led to the accumulation of highest beta-caryophyllene (a sesquiterpene) in *Cannabis* (cv. CBD kush) floral tissue, which was twice than that of non-PGPR inoculation (Lyu et al., 2023). An endophytic fungus *Piriformospora indica* inoculated in *Stevia rebaudiana* plant stimulates the production of steviol glycosides (Kilam et al., 2017; Chu et al., 2026).

Inoculation of Grape plants with two PGPR bacteria, *Bacillus licheniformis* and *Pseudomonas fluorescens* promote the synthesis of terpenes (Salomon et al., 2014). *Bacillus pumilus* G5 has been reported to increase glycyrrhizic acid content thereby protecting *Glycyrrhiza uralensis* seedlings from adverse effects of drought (Zhang et al., 2020). A similar result was observed when potted plants were inoculated with *Rhizobium rhizolycopersici* GUH21 under high moisture and low temperature conditions (Liu et al., 2023).

Two endophytic fungi *Chaetomium globosum* D38 and *Trichoderma atroviride* D16, which generally inhabit the roots of *Salvia miltiorrhiza*, increase tanshinone accumulation (Zhai et al., 2018). Endophytic bacteria like *Bacillus subtilis, Burkholderia gladioli, Trichoderma hamatum, Neopestalotiopsis* sp., and *Fusarium decemcellulare* promote plant growth and triterpenoid content in *Schsandra sphenanthera* (You et al., 2021). When the roots of *Astragalus mongholicus* are inoculated with a specific bacterial consortium it enhances the production of terpenoids like astragaloside IV (Lin et al., 2022). Additionally, *Stenotrophomonas* bacteria in rhizosphere were found to be directly correlated with astragaloside content indicating their influence on the accumulation process (Li et al., 2021).

Similarly, inoculation of *Trichoderma asperellum* ACC30536 in *Artemisia annua* resulted in increased leaf artemisinin production and improved soil nutrition (Zhai et al., 2019). In another study, a consortium of four bacterial endophytes inoculated into *A. annua* plants enhanced artemisinin yield by approximately 65.8% compared to the uninoculated control (Tripathi et al., 2020) The interaction of root endophyte *Serendipita indica* with *Solanum lycopersicum* induces expression of genes responsible for biosynthesis of specific terpene in both roots and leaves (Ntana et al., 2022).

Two fungal pathogens, *Fusarium solani* and *Fusarium oxysporum* increase ginsenoside (a terpenoid) content in *Panax quinquefolius* (Jiao et al., 2015). It indicates that even pathogenic fungi play important roles in promoting plant growth and terpenoid accumulation. Three fungal endophytes i.e., *Fusarium redolens, Phialemoniopsis cornearis*, and *Macrophomina pseudophaseolina* regulate biosynthesis of forskolin in *Coleus forskohlii* (Mastan et al., 2019). *Streptomyces* strain strep-4 from rhizosphere soil promote the synthesis and accumulation of monoterpenes by inducing systemic resistance in *Citrus reticulata* (Su et al., 2023). A study reported the presence of several terpenoid metabolic pathways in the seed microbiota of *Salvia miltiorrhiza* (Chen et al., 2018). These studies collectively indicate the stimulatory role of microbial consortia either through natural or synthetic means in inducing and promoting terpenoid biosynthesis in many plants.

#### 4.1.2 Phenolic compounds

They represent a large class of secondary metabolites that are widespread in plants and play crucial roles in various physiological processes such as plant quality, coloring, flavor and stress resistance. These compounds exhibit diverse properties including antioxidant, antimicrobial, anticancer, anti-inflammatory, anti-atherogenic, anti-allergenic activities (Manach et al., 2005). Additionally, these secondary metabolites are significant for plant defense mechanisms. They include various compounds such as flavonoids, phenolic acids, tannins, stilbenes, lignans and colored anthocyanins (Babbar et al., 2014).

In a study of *Papaver somniferum* L. inoculated with a consortium of *Marmoricola* sp., and *Acinetobacter* sp., morphine yield was found to be enhanced (Ray et al., 2019). Inoculation of *Ralstonia pickettii* and *Brevibacillus invocatus* raised flavonoid and phenolic content in sunflower (cv. Parsun3) grown under polluted conditions (Urooj et al., 2022).

An endophytic bacterium *Achromobacter* sp. associated with *Polygonum cuspidatum* promotes polydatin synthesis in roots (Zhang et al., 2020). *Methylobacterium* sp. modulates the synthesis and accumulation of phytometabolites related to flavor and metabolizes volatile organic compounds (Brader et al., 2014). Similarly, the two rhizosphere bacteria *norank-o-Gaiellales* and *norank-f-AKYG1722* promoted the accumulation of total sugars and flavonoids in the fruits of *Lycium barbarum* (Liu et al., 2022a,b). Inoculation of PGPR with *Lycopersicum esculentum* resulted in enhanced levels of total phenols, flavonoids, anthocyanin and polyphenol contents (Khanna et al., 2019). Lignin-derived compounds were found to increased when *Serendipita indica* was inoculated in to *Solanum lycopersicum* (Ntana et al., 2022). Another study reported that *Phyllobacterium* sp. and *Inquilinus* sp. in endosphere are related to calycosin content (Li et al., 2021).

Endophytic fungi are also known to influence the accumulation of phenolic compounds (Kocer et al., 2026). The fungus *Paraconiothyrium variabile* metabolizes glycosylated flavonoids in *Cephalotaxus harringtonia* leading to the production of aglycones (Tian et al., 2014). Colonization of *Ocimum basilicum* by the arbuscular fungus *Glomus mosseae* confers a bioprotective effect against *Fusarium oxysporum* by promoting the synthesis of phenolic substances rosmarinic acid (Zhang et al., 2013). Similarly, colonization of *O. basilicum* with *Glomus intraradices* increases total anthocyanins contents, whereas it increases the total phenolic acids in *Arnica montana* leaves and the concentration of phenolic substances in *Echinacea purpurea* (Jurkiewicz et al., 2010). Six arbuscular mycorrhizal fungus (AMF) species like *Glomus clarum, G. etunicatum, G. fasciculatum, Gigaspora* sp.*, G. mossae*, and *Acaulospora* sp. promote synthesis of polyphenol, flavonoids, anthocyanin in *Dioscorea* sp. under field cultivation condition (Lu et al., 2015).

The *G. versiforme* inoculation significantly increased both free phenolic and cell wall bound phenolics in roots of *Poncirus trifoliata* (Li et al., 2015). Inoculation of native AMFs to *Salvia militiorrhiza* facilitated significant accumulation of phenolic acids (Wu et al., 2021). The AMF association of *Rhizophagus irregularis* in *Vitis* sp. (Grape vine) stimulates phenylpropanoid synthesis and stilbenoid production by regulating the key genes with response to downey mildew and gray mold infection (Bruisson et al., 2016). *Houttuynia cordata Thunb*. inoculated with *Pseudomonas fluorescens* increased plant growth, chlorogenic acid, rutin, and quercetin content (Wang et al., 2024).

#### 4.1.3 Alkaloids

Alkaloids are a group of naturally occurring compounds containing one or more nitrogen atoms, typically in a heterocyclic ring (amine as functional group). They often have pharmacological ‘effects and primarily protect plants from predators and pathogens. Alkaloids exhibit antiproliferative, antibacterial, anesthetic, cardioprotective, and anti-inflammatory properties (Kurek, 2019).

The colonization of tomato plants by endophytic fungus *Beauveria* sp. AUMC 15401 increased alkaloid production compared to its control (Salem et al., 2022). Inoculation of *Leucojum aestivum* with the endophytic bacteria *Paenibacillus lautus* increased levels of galanthamine and lycorine alkaloids (Ptak et al., 2022).

In a study, PGPR bacterium N5.18, *Stenotrophomonas maltophilia* increased the total alkaloid content specifically morphine, codeine and oripavine in *Opium poppy* (Bonilla et al., 2014). A consortium of *Rhizophagus intraradices, Gigaspora margarita*, and *Claroideoglomus etunicatum* inoculation in to *Catharanthus roseus* increased the synthesis of ajmalicine (Monnerat et al., 2018) while *C. etunicatum* increased the biosynthesis of nicotine, anabasine, and nornicotine in roots and leaves of tobacco (de Andrade et al., 2013).

*Pseudomonas putida* and *Agrobacterium rhizogenes* enhanced accumulation of pyrroloquinazoline alkaloids in the hairy roots of *Adhatoda vasica* (Singh et al., 2022b,a). Similarly, inoculation with nine fungal endophytes promoted the accumulation of total alkaloids in *Lycoris radiata* plants (Zhou et al., 2020). Synthesis of benzylisoquinoline (BIA) alkaloids was enhanced in *Opium poppy* plants when inoculated with *Acinetobacte*r SB1B (Pandey et al., 2016a). Additionally, *Aspergillus sydowii* PET-2, a fungus derived from Pu-erh tea is considered an effective strain for caffeine metabolism and increasing theophylline content in *Camellia sinensis* (Zhou et al., 2019). Colonization of *Bacillus* sp. caused systemic exudation of acyl sugars in tomato plants (Korenblum et al., 2020). The microbiome of bacteria *Echinacea purpurea* affect alkamide synthesis in plants (Maggini et al., 2019). Likewise, *Glomus fasciculatum* was reported to increase allicin content in *Allium sativum* (Borde et al., 2009).

The accumulation of matrine, oxymatrine, and sophoridine in the roots of *Sophora flavescens* correlates with soil rhizosphere bacterial community predominantly by *Actinobacteria* sp. and *Chloroflexi* sp. (Chen et al., 2021). All these reports highlight the crucial role of plant growth promoting microorganisms in accumulation of alkaloids.

#### 4.1.4 Essential oils

Essential oils are potent and concentrated hydrophobic liquids obtained from various plants possessing diverse biological activities such as antimicrobial, antihelminthic, antiviral, antioxidant, anti-inflammatory, insecticidal, and larvicidal effects. Their versatility and diverse benefits make them valuable compounds with potential applications in various fields, from medicine to pest control and more (Dhifi et al., 2016).

PGPRs like *Pseudomonas fluorescens, Bacillus subtilis*, and *Azospirillum brasilense* increased essential oil content in *Origanum x majoricum* (Banchio et al., 2010). Similarly, the dual inoculation of *Halomonas desertis* G11 and *Oceanobacillus iheyensis* E9 in *Pelargonium graveolens* plants led to the highest production of essential oil in leaves, with an increase of 68.96% as compared to the control plant (Riahi et al., 2020).

In *Ocimum basilicum* and *Satureja hortensis* inoculation of *Glomus mosseae*, increased the synthesis of essential oil content by approximately 39% and 25%, respectively, as compared to plants treated with mineral fertilizer only (Khalediyan et al., 2021). Three arbuscular fungi, *Glomus mosseae, G. fasciculatum*, and *G. intraradices*, enhanced the production of essential oil and plant growth in *Ocimum basilicum* (Zolfaghari et al., 2013). *Pseudomonas fluorescens* and *Azospirillum brasilence* together enhanced essential oil levels in Marigold by about 70% as compared to the control (del Rosario Cappellari et al., 2013). Similarly, in *Lippia alba*, inoculation with *Claroideoglomus etunicatum* and *Fuscutata heterogama* positively influenced the composition of essential oils (Palhares Neto et al., 2023). *Glomus etunicatum* in *Pogostemon cablin* plants and *G. versiforme* in tobacco plants are also reported to increase the synthesis of essential oil production (Begum et al., 2021). The combined application of PGPR and AMF increased the essential oil yield in *Melissa officinalis* leaves (Eshaghi Gorgi et al., 2022). The study involving *Trichoderma harzianum* and *Brevibacterium halotolerans* in *Mentha arvensis* plant under greenhouse and field conditions, showed enhanced biosynthesis of essential oil (Singh et al., 2019).

In addition to the aforementioned metabolites, plants also produce other secondary metabolites such as polysaccharides, benzoxazines, anthraquinones, and naphthoquinones, etc. which are also indirectly induced by microbial interactions. These findings highlight vast potential of microbes in enhancing plant secondary metabolites biosynthesis (Table 2).

**Table 2:** Microorganisms influencing plant secondary metabolite biosynthesis.

Microorganism/ consortium	Host plant	Secondary metabolite	Type	Response	References
*Mucilaginibacter* sp. *Pseudomonas* sp.	*Cannabis sativa* L.	Total terpene	Terpenoid	Approximately 23% increase by *Mucilaginibacter* sp. and 18% by *Pseudomonas* sp. compared to control	(Lyu et al., 2023)
*Streptomyces strep*-4	*Citrus reticulata*	Monoterpene	Terpenoid	Promotes accumulation, increased fruit quality	(Su et al., 2023)
*Rhodoplanes* sp., *Pseudoxanthomonas* sp., *Erwinia* sp., *Sphingobium* sp., *Apiotrichum* sp., *Solicoccozyma* sp., *Alternaria* sp., *Vishniacozyma* sp.	*Paeonia lactiflora*	Paeoniflorin	Terpenoid	Promote accumulation	(Sun et al., 2022)
*Glomus bagyarajii*	*Coleus forskohlii*	Forskolin	Terpenoid	Content increased in root significantly	(Sailo and Bagyaraj, 2005)
*Rhizophagus irregularis*	*Plantago lanceolata*	Catapol	Terpenoid	Promotes accumulation	(Schweiger et al., 2014)
*Bacillus* sp.	Wheat (*Triticum aestivum*)	Geranyl acetate	Terpenoid	Promoted synthesis	(Timmusk et al., 2014)
*Glomus intraradices, G. mosseae, G. versiforme*	*Salvia miltiorrhiza*	Tanshione	Terpenoid	Increased synthesis of metabolite	(Yang et al., 2017)
*G. versiforme*	*Salvia miltiorrhiza*	Salvianolic acid B	Phenolic	Increased accumulation	(Yang et al., 2017)
*Acinetobacter pitti, Bacillus subtilis, B. licheniformis, Burkholderia* sp.	*Artemisia annua*	Artemisinin	Tepenoid	65.8% increase compared to uninoculated control	(Tripathi et al., 2020)
*Pseudomonas fluorescens, Bacillus subtilis, Azospirillum brasilense*	Oregano (*Origanum x majoricum*)	Thymol Carvacrol Sabinene-hydrate Y-terpinene	Terpenoid	Increased the terpenoid content and essential oil production	(Banchio et al., 2010)
*Piriformospora indica*	*Stevia rebaudiana*	Steviol glycosides	Terpenoid	Promoted accumulation	(Kilam et al., 2017)
*Bacillus pumilus* G5	*Glycyrrhiza uralensis*	Glycyrrhizic acid	Terpenoid	Accumulation stimulated	(Liu et al., 2023)
*Bacillus subtilis, Burkholderia gladioli, Trichoderma hamatum, Neopestalotiosis* sp.*, Fusarium decemcellulare*	*Schsandra sphenanthera*	Triterpene	Terpenoid	Promoted accumulation compared to control	(You et al., 2021)
*Stenotrophomonas* sp.	*Astragalus mongholicus*	Astragaloside	Terpenoid	Synthesis of metabolite enhanced	(Li et al., 2021)
*Rhizoglomus irregulare, Funneliformis mosseae*	*Lactuca sativa var. crispa*	Total phenol and anthocyanin	Phenolic	Total phenol and anthocyanin content increased compared to control in leaf tissue	(Avio et al., 2018)
*Cloideoglomus etunicatum, Funneliformis mosseae, Rhizophagus intraradices*	*Cassia italica Mill*	Total phenol	Phenolic	Increased phenol content ensuring better growth under stressed condition	(Hashem et al., 2016)
*Pseudomonas fluorescens* N 21.4	Soybean seeds	Isoflavones	Phenolic	Increased synthesis of metabolite, induced systemic resistance	(Martin-Rivilla et al., 2020)
*Bacillus subtilis* (BHHU100)*, Pseudomonas aeruginosa* (PJHU15)*, Trichoderma harzianum* (TNHU27)	Pea	Gallic acid, Chlorogenic acid, Shikimic acid, Syringic acid, p-Coumaric acid, Salicylic acid	Phenolic	Accumulation increased, resistance to *Sclerotinia sclerotiorum*	(Jain et al., 2015)
*Enterobacter ludwigi* EnVs6	Grape	Kaempferol	Phenolic	Facilitates endosphere colonization	(Lòpez-Fernàndez et al., 2016)
*Serratia marcescens*	Betel vine (*Piper betle*)	Ferulic acid	Phenolics	Accumulation enhanced, resistance to *Phytopthora nicotianae*	(Lòpez-Fernàndez et al., 2016)
*Paenibacillus* sp. *Pseudomonas* sp.	*Fraxinus excelsior*	Total phenols, flavonoids, and carotenoids	Phenolic	Promoted accumulation	(Striganavičiute et al., 2021)
*Streptomyces lydicus*	Tomato	Salicylic acid	Phenolic	Increased content, plant growth	(Wu et al., 2018)
*Stenotrophomonas maltophilia, Curtobacterium* sp.	Soybean	Daidzen	Phenolics	Resistance to *Xanthomonas axonopodis*	(Algar et al., 2014)
*Rhizobium leguminosarum bv. phaseoli, R. leguminosarum bv. trifolii*	Rice	Ferulic acid, Gallic acid	Phenolic	Accumulation promoted, resistance to *Rhizoctonia solani*	(Mishra et al., 2006; Chamam et al., 2013)
*Rhizophagus irregularis*	Olive	Flavonoids	Phenolic	Increase in total phenol and flavonoids	(Mechri et al., 2015)
*Pseudomonas putida*	*Adhatoda vasica*	Pyroquinolinealkaloids	Alkaloid	Increased accumulation in hairy roots	(Singh et al., 2022b,a)
*Bacillus simplex* (SmebS45)	Soybean	Piperine	Alkaloid	Stimulated accumulation, nematicidal activity	(Kang et al., 2018)
*Bradyrhizobium* sp.	Rattlepods (*Crotalaria* sp.)	Monocrotaline	Alkaloid	Accumulation increased, antiherbivore defense	(Irmer et al., 2015)
AMF	*Catharanthus roseus*	Vincarine	Alkaloid	Content increased >100%	(De la Rosa-Mera et al., 2011)
*Claroideoglomus etunicatum*	Tomato (*Lycopersicon esculentum*)	Tomatidine	Alkaloid	Increased content, salt tolerance	(Rivero et al., 2018)
*Rhizophagus irregularis*	Ragwort (*Senecio jacobaea*)	Senecionine	Alkaloid	Anti-herbivore defense	(Hill et al., 2018)
*Norank-f-Anaerolineaceae*	*Lycium barbarum*	Betaine	Alkaloid	Promoted accumulation	(Liu et al., 2022a)
*Actinobacteria* sp.	*Sophora flavescens*	Oxymatrine, matrine	Alkaloid	Significant correlation	(Chen et al., 2021)
Microbiome	*Vetiveria zizanioides*	Oil content	Essential oil	Affected composition of essential oil	(del Rosario Cappellari et al., 2013)
*Glomus etunicatum*	*Pogostemon cablin*	Oil content	Essential oil	Increased the content	(Arpana et al., 2008)
*AMF* (*Diversispora tortuosa*)	*Matricaria chamomilla*	Oil content	Essential oil	Promoted accumulation	(de Almeida et al., 2020)
PGPR+AMF	*Melissa officinalis*	Oil content	Essential oil	Increased metabolite	(Eshaghi Gorgi et al., 2022)
*Glomus etunicatum*	*Foeniculum vulgare*	Oil content	Essential oil	Increased metabolite	(Kapoor et al., 2004)
*Rhizophagus irregularis*	*Anethum graveolens*	Oil content	Essential oil	Increased accumulation	(Rydlová et al., 2016)
*Claroideoglomus esculentum*	*Lippia alba*	Oil content	Essential oil	Composition of Eos increased	(Palhares Neto et al., 2023)
*Pseudomonas putida* SJ04	*Mentha piperita*	Oil content	Essential oil	Promoted accumulation	(del Rosario Cappellari et al., 2020)
*Glomus versiforme*	*Nicotiana tabacum*	Oil content	Essential oil	Promoted accumulation	(Begum et al., 2021)
*Bacillus* sp.	*Cannabis sativa*	THC and CBD	Others	Content positively affected	(Pagnani et al., 2018)
*Pseudomonas* sp. *Azospirillum* sp.	Maize	Benzoxazinoids	Others	Multiple effect on plant health and development along with enhanced accumulation	(Couillerot et al., 2013)
*Aspergillus* sp.	*Rumex gmelini* Turcz	Emodin, chrysophanol, and resveratrol	Others	Increased the content	(Ding et al., 2018)
*Allorhizobium* sp., *Neorhizobium* sp., *Pararhizobium* sp., *Rhizobium* and *Labrys* sp.	*Alkanna tinctoria*	Acetylakannin, alkanin, napthoquinone	Others	Synthesis stimulated	(Csorba et al., 2022)
*Fusarium oxysporum*	*Dioscorea zingiberensis*	Diosgenin	Others	Enhanced synthesis	(Li et al., 2011)
*norank-f-*(*AKYG1722*), *norank-o-Gaiellales*	*Lycium barbarum*	Polysaccharides and sugars	Others	Promote accumulation in fruits	(Liu et al., 2022a)
*Azospirillum brasilense, Bacillus subtilis Pseudomonas fluorescens*	*Solanum melongena* L.	Nasunin, Chlorogenic acid, and solasodine	Phenolic, flavonoid	Enhanced synthesis	(Bhattarai, 2025)

## 5 Microbial regulation in plant secondary metabolite biosynthesis

### 5.1 Regulation through plant hormone response

The accumulation of secondary metabolites in plants is induced by several phytohormones such as auxin, gibberellins (GA), jasmonic acid (JA), abscisic acid (ABA), and salicylic acid (SA; Zhang et al., 2023). These hormones are the primary mediators in plant secondary metabolite synthesis influencing the movement, accumulation and release of secondary metabolites (Lv et al., 2021).

Microorganisms induce the synthesis of secondary metabolites in plants through two primary mechanisms. First, some microorganisms synthesize phytohormones to promote plant growth and photosynthesis (Marks et al., 2025). Photosynthesis produces several organic compounds and generates NADPH. This NADPH indirectly affects secondary metabolite synthesis in plants because the enzyme cytochrome P450 monooxygenase (CYPs), found on the endoplasmic reticulum is involved in the synthesis process. The energy required for this comes from NADPH electron transport. In plants, CYPs constitute the third largest multigene family and significantly contribute to the accumulation of diverse metabolites (Guo et al., 2016). CYP 72 has been reported to be involved in steviol biosynthesis and gibberellin catabolism indicating their close relationship in secondary metabolism pathways and hormone metabolism (Nomura et al., 2013).

Various studies have found that JA, SA, GA, and ABA have strong correlations with secondary metabolites in plants (Cheng et al., 2019). The endophyte *Pseudomonas fluorescens* ALEB7B increases sesquiterpenoids accumulation in *Atractyoles lancea* by producing Indole-3-acetic acid which in turn enhances root development, plant growth, vigor and photosynthesis. Additionally, exogenous application of IAA increases other secondary metabolites (Zhou et al., 2018). SA has been reported to activate flavonol biosynthetic pathway in *Dendrobium officinale* (Yu et al., 2021). The exogenous application of ABA on medicinal plants *Glycyrrhiza uralensis* and *Salvia miltiorrhiza* significantly enhances the accumulation of secondary metabolites (Ptak et al., 2022). *Streptomyces* rhizobacteria enhance root development and photosynthesis in plants and play a significant role in secondary metabolism (Salla et al., 2014).

**Figure 2:**
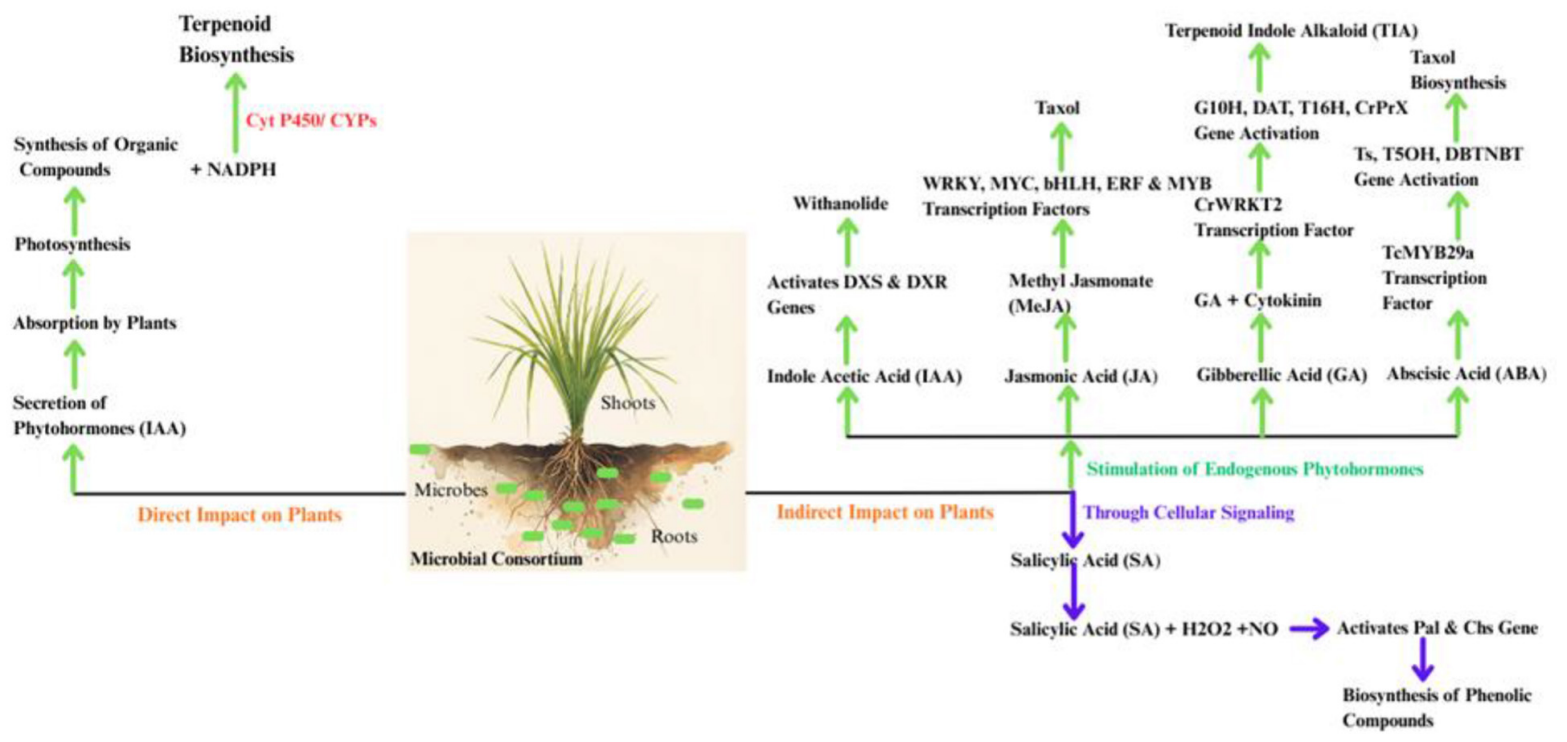
Microbial regulation of secondary metabolites synthesis through plant hormone. Microbes secrete phytohormones promoting plant growth and development and trigger biosynthesis of terpenoids via key enzymatic pathways (direct impact). Beneficial microorganisms stimulate phytohormone production in plants, activating transcription factors and enzymes that drive secondary metabolite biosynthesis and these phytohormones in conjunction with nitic oxide and hydrogen peroxide interactively modulate biosynthesis of secondary metabolites in plants (indirect impact).

Secondly microorganisms enhance the expression of key genes and transcription factors by influencing endogenous plant hormone levels, thus regulating secondary metabolism. Five bacterial species isolated from *Withania somnifera* namely *Bacillus muralis, Bacillus megaterium, Pseudomonas* sp.*, Streptomyces* sp., *and Pantoea* sp. regulate endogenous auxin content by synthesizing IAA. This in turn, activates the expression of methylerythriol phosphate pathway genes 1-deoxy-D-xylose 5-phosphate synthase (DXS) and 1-deoxy-D-xylose-5-phosphate reductoisomerase (DXR) increasing the synthesis of withanolide and withferin-A (Pandey et al., 2018; Figure 2).

Phytohormone Jasmonic acid regulates the synthesis and accumulation of secondary metabolites in several medicinal plants (Rahimi et al., 2014). Colonization of an endophytic fungus in *Taxus chinennsis* enhances phytohormone levels. Interaction of phytohormones with JA signaling process stimulates the alteration of transcription factor thereby regulating the synthesis of Taxol in plants (Cao et al., 2022).

JA and ABA have been reported to regulate the synthesis of anthocyanins and phenolic acids influenced by mycorrhizal colonization (Adolfsson et al., 2017). Inoculation of *Pseudomonas fluorescens* activates the JA and ethylene in addition to producing cytokinin and GA. This in turn increases the expression of transcription factor CrWRKT2, leading to expression of geraniol-10-hydroxylase (G10H), deacetylvindoline-4-O-acetyltransferase (DAT), tabersonine 16-hydroxylase (T16H) and final gene of synthetic pathway *Catharanthus roseus* apolplastic peroxidase (CrPRX) in the terpenoid indole alkaloids (TIA) biosynthesis pathway of *Catharanthus roseus* (Ahmadzadeh et al., 2022).

In addition to the mechanisms described above, hydrogen peroxide (H_2_O_2_) and nitric oxide (NO) affect the synthesis of plant secondary metabolites as they interact with various phytohormones signaling pathways. Both molecules have been reported to increase the production of volatile compounds in some aromatic plants (Fang et al., 2009). AMF activate phenylalanine ammonia lyase (PAL), the key enzyme of phenyl alanine pathway by elevating the levels of SA, H_2_O_2_, and NO leading to enhanced production of phenolic compounds in clover roots (Zhang et al., 2013; Zhu et al., 2015). In navel orange plants, it has been reported that H_2_O_2_ plays a key role in systemic increase in PAL activity and develops immunity against citrus canker disease induced by SA (Wang and Liu, 2012).

### 5.2 Regulation through nutritional response

Microorganisms influence the synthesis of plant secondary metabolites by enhancing the absorption of plant nutrients (Hashemabadi et al., 2018). Improved plant nutrition influences growth and photosynthetic rates, and these photosynthates, in turn, provide precursors for their synthesis (Santoro et al., 2016; Liu et al., 2022a).

Although phosphorus is relatively insoluble in soil, it plays a crucial role in key metabolic processes, such as nitrogen and carbon metabolism and photosynthesis, thus positively impacting crop growth and development. AMF plays an important role in plant nutrient mineralization. Plant roots associated with AMF secrete organic acids and enzymes like phosphatases, facilitating phosphorus solubilization. Additionally, AMF increases the surface area of roots and forms arbuscules in cortical region aiding nutrient exchange, boosting growth and photosynthetic efficiency. Microorganisms such as *Bacillus* sp. and *Pseudomonas* sp. have been reported to facilitate phosphorous solubilization and absorption by plant roots (Singh et al., 2022a,b). Phosphorous enhances pyrophosphate concentration containing high-energy phosphate bonds such as IPP (isopentenyl diphosphate) and DMAPP (dimethylallyl diphosphate; Kapoor et al., 2004; Zubek et al., 2010). Limitation of phosphorous leads to membrane destabilization causing increased isoprene emission and thus improved uptake reduces this effect (Kobayashi et al., 2009). Hence, phosphorous plays an important role in the biosynthesis of isoprenoids which requires acetyl-CoA, ATP, and NADPH through Mevalonate (MVA) pathway and glyceraldehyde phosphate and pyruvate in non-MVA or the Methylerythritol phosphate (MEP) pathway. This shows that enhancing terpene-derived essential oils strongly correlates with phosphorous uptake by the plant. Thus, phosphorous serves as one of the direct promoters for terpenoid synthesis by AMF and PGPRs (Khalediyan et al., 2021; Ran et al., 2022).

**Figure 3:**
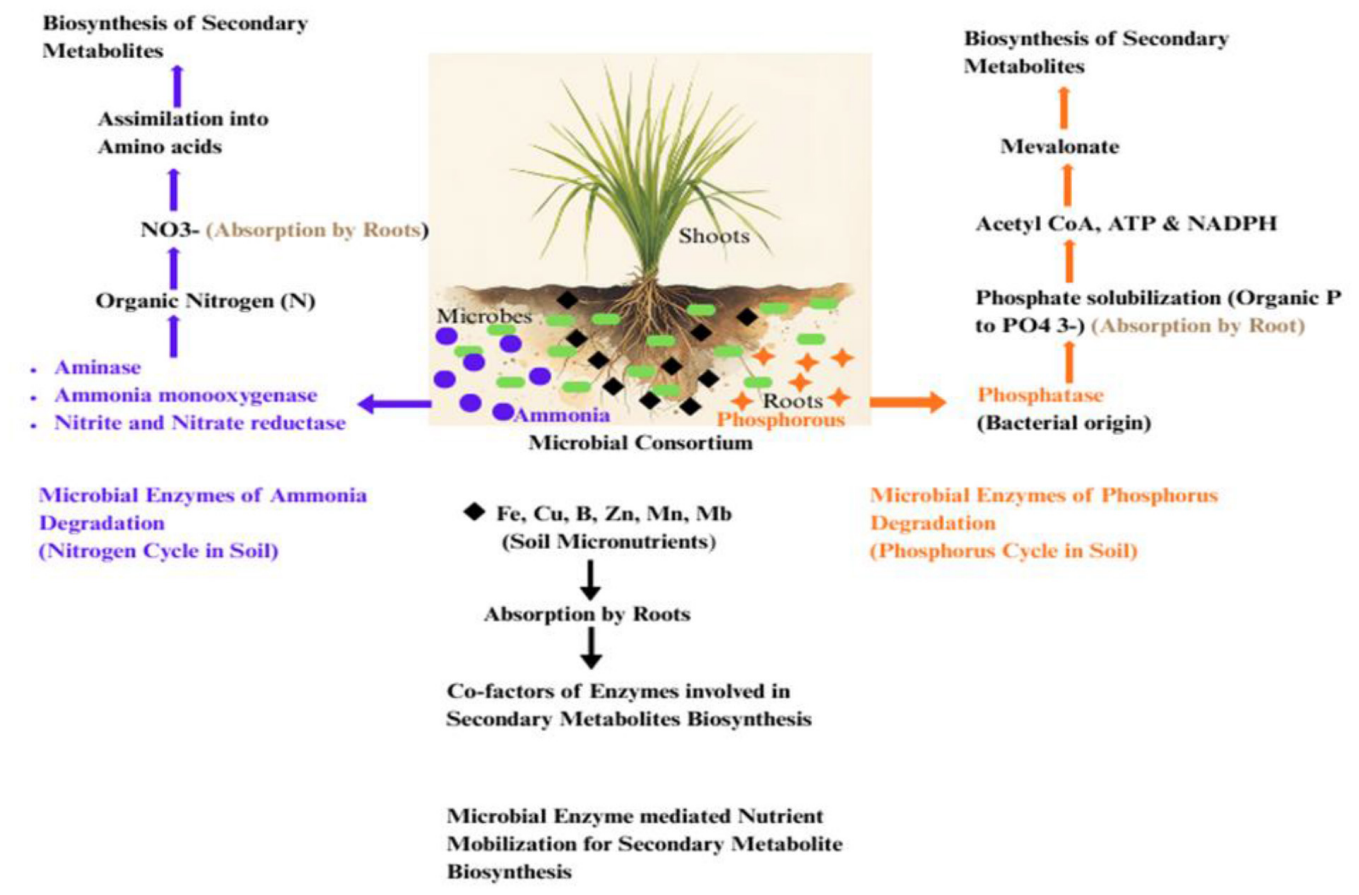
Nutrient-mediated regulation of secondary metabolites synthesis. Beneficial microbes facilitate nutrient mobilization boosting plant nutrition, growth and provide metabolic precursors and energy required for secondary metabolite synthesis.

Nitrogen is an important component of amino acids, which are key precursors to alkaloids and its deficiency can adversely affect terpenoid synthesis. The extraradical hyphae of AMF absorb ${\text{NH}}_{4}^{+}$ from the soil and convert inorganic nitrogen in to amino acids using glutamine synthetase. These amino acids are then converted back to urea and ammonia by arginase and urease, respectively, making them accessible to the host plant. Simultaneously, they also provide organic nitrogen by decomposing organic matter in the soil (Tao et al., 2019). Additionally, some PGPRs and AMF enhance uptake and availability of nitrogen to plants roots through nitrogen fixation. For instance, *Phomopsis liquidambris* colonization in peanut roots improves nodulation and N_2_ fixation (Xie et al., 2019a,b). Plants also employ nitrifying bacteria to convert organic nitrogen in to ${\text{NO}}_{3}^{-}$ through NRT1.1B (nitrate transporter and sensor). Studies showed that the upregulation of plant transcripts for gene coding for ${\text{NO}}_{3}^{-}$ and ${\text{NH}}_{4}^{+}$ occurs following AMF colonization, with similar upregulation in fungal transporters (Koegel et al., 2017). Nitrogen, as NADPH is required for the biosynthesis of terpenoids and AMF increase nitrogen uptake in essential oil-bearing plants. A positive correlation has been observed between nitrogen and betaine content in *Lycium barbarum* (Liu et al., 2022a,b; Figure 3).

Nutrient absorption process significantly impacts the plant's secondary metabolite spectrum. An increased nitrogen supply has been reported to decrease the level of carbon based secondary metabolites like polyphenol. According to the Growth Differentiation Balance Hypothesis (GDBH), growth and differentiation (synthesis of carbon-based compounds like terpenoids and phenolics) are determined by the availability of carbon and nutrients in the environment. Increased nitrogen content favors growth over secondary metabolite production, but when nitrogen becomes limited or optimal, it leads to an increased accumulation of terpenoids or phenolics. Therefore, a relatively greater C:N ratio allocates more carbon toward the plant growth, while a lower ratio promotes the production of carbon-based compounds such as glycyrrhizin (Xie et al., 2018; Yu et al., 2021). Secondary metabolites in plants are also affected by micronutrient uptake processes, directly or indirectly, as most micronutrients are cofactors for several enzymes involved in their metabolite biosynthetic pathways. Rhizosphere or endophytic microorganisms absorb micronutrients and translocate them to plants through various mechanisms such as by iron carriers to provide iron to the plant. This in turn, affects the secretion of root exudates enriched with organic acids to increase zinc availability to the plant (Singh et al., 2022a,b). Additionally, plant glandular trichomes present on different parts of plant help in the synthesis and accumulation of several natural products.

### 5.3 Regulation through key enzymes and gene expression

The synthesis of secondary metabolites can be regulated by key enzymes and the expression of genes involved in the biosynthetic pathway. One such key enzyme is 3-hydroxy-3-methylglutaryl coenzyme-A reductase (HMGR), which plays a pivotal role in the MVA pathway, contributing to the biosynthesis of sesquiterpenes.

Colonization of endophytic fungi *Chaetomium globosum* D38 with *S. miltiorrhiza* hairy roots stimulates the expression of several enzymes, such as HMGR and DXR (deoxyd-xylulose 5-phosphate reductoisomerase), in the MVA and MEP pathways, respectively. Similarly, the expression levels of geranylgeranyl diphosphate synthase (GGPPS), copalyl diphosphate synthase (CPSs) and kaurene synthase-like (KSL) are elevated leading to the biosynthesis of tanshinone (Zhai et al., 2018).

Inoculation of *Glycyrrhiza uralensis* with *Rhizophagus irregularis* augments the expression of glycyrrhizin synthesis genes SQS1 (squalene synthase), β-AS (amyrin synthase), CYP88D6 (cytochrome P450 monooxygenase 88D6) and CYP72A154 resulting in enhanced glycyrrhizic acid. The expression of UGAT (UDP dependent glucuronosyltransferases) and CHS (Chalcone synthase gene) increases liquiritin content (Xie et al., 2018).

*Alcaligenes faecalis* MH998155 in *Coleus forskohlii* plants upregulates several diterpene synthases enzymes (CfTPS1, CfTPS2, CfTPS3, and CfTPS4) leading to enhanced forskolin content in its biosynthetic pathway. Another study on the same plant showed increased expression of CfTPS2 following inoculation with *Macrophomina pseudophaseolina*. Similar results were noted with inoculation of *Trichoderma viride*. Moreover, the combined application of RF1 (*Fusarium redolens*1) and TV1 (*Trichoderma viride*1) resulted in elevated expression of CfTPS2, CfTPS4, CfCYP&6AH15, and CfACT1-8 (Mastan et al., 2021).

Phenylalanine ammonia-lyase1 (Pal1) is an essential enzyme in phenylpropanoid bio synthesis, transforming L-phenylalanine to ammonia and trans-cinnamic acid, contributing to accumulation of phenolic compounds in plants (Rahimi et al., 2021; Ortiz and Sansinenea, 2023). Pal1 expression was higher in *Cydonia oblonga* Mill. *cv. Isfahan* colonized by *Funneliformis mossae* and *R. intraradices*. The expression of the tyrosine aminotransferase (TAT), hydroxyphenylpyruvate reductase (HPPR) and *p*-coumaroylmangiferyl 3'-hydroxylase (CS3'H iso1) genes involved in the biosynthesis of rosmarinic acid (RA) were elevated when *Sinorhizobium meliloti* TSA41, and *Streptomyces* sp. W43N were inoculated in basil leaves (Battini et al., 2016).

**Figure 4:**
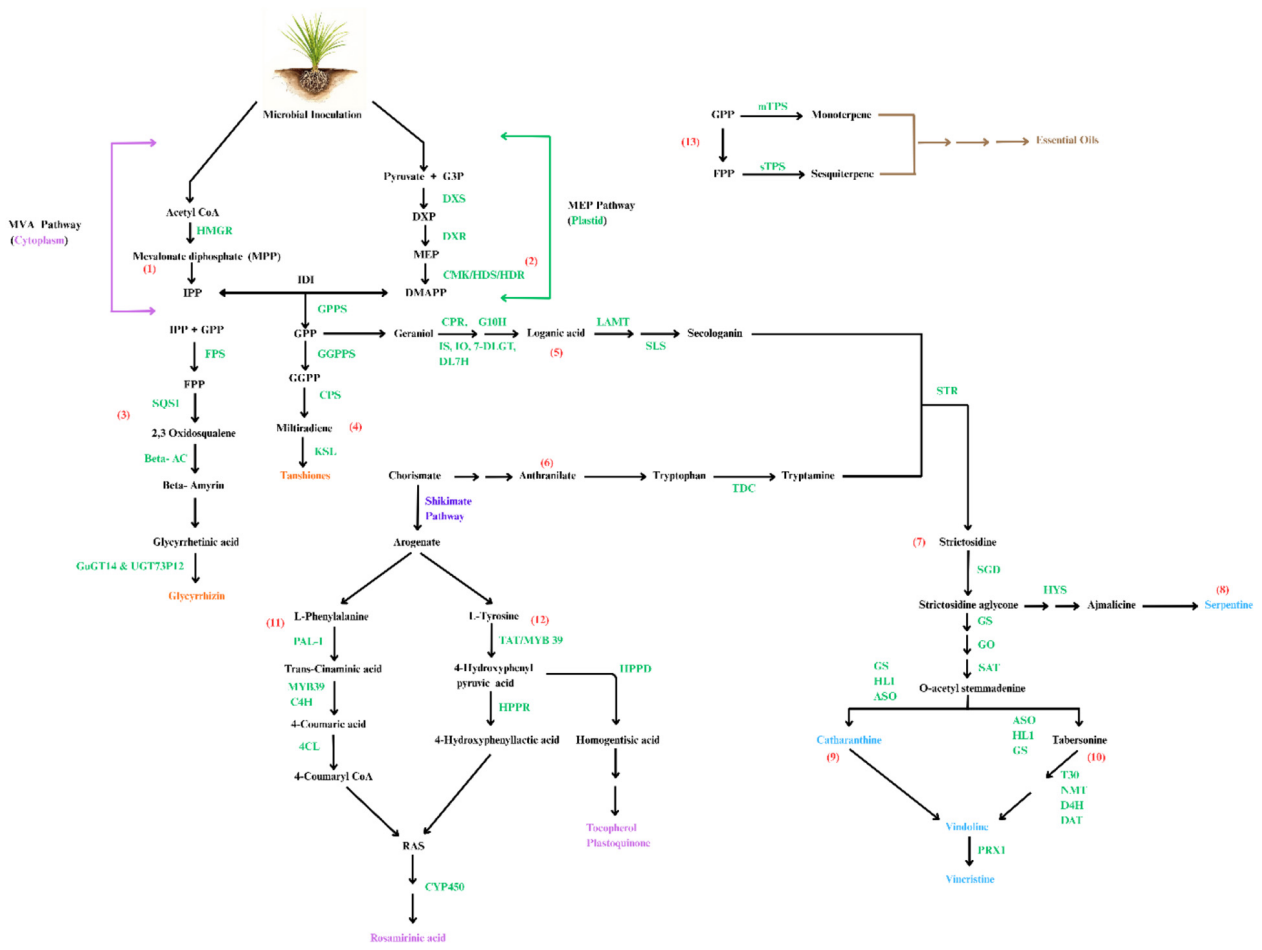
Microbial regulatory mechanism of secondary metabolite synthesis through key enzymes and genes. The biosynthesis of secondary metabolites involves two pathways: (1) MVA (Mevalonate) Pathway and (2) MEP (Methylerythritol phosphate) Pathway, which generate the precursors isopentenyl pyrophosphate (IPP), and its isomer dimethylallyl pyrophosphate (DMAPP), respectively. IPP and DMAPP combine to form geranyl pyrophosphate (GPP), a precursor for various terpenoids, (3) which is then converted to farnesyl pyrophosphate (FPP), a key intermediate in the biosynthesis of glycyrrhizin. (4) Tanshione is synthesized from GPP via geranylgeranyl pyrophosphate (GGPP). (5) Secologanin obtained from GPP combines with (6) tryptamine derived from the chorismate to form Strictosidine in number of steps regulated by several genes and enzymes. (7) Strictosidine is a key intermediate in biosynthesis of various alkaloids such as O-acetylstemmadenine and (8) Serpentine which is regulated by enzyme hetero yohimbine synthase (HYS). (9) O-acetylstemmadenine is converted to catharanthine. (10) Vindoline derived from tabersonine combines with catharanthine to form vincristine, a valuable alkaloid. (11) Chorismate, key intermediate in shikimate pathway is transformed to phenylalanine and tyrosine through separate enzymatic steps. (12) These amino acids serve as precursors for biosynthesis of rosamirinic acid, a phenolic compound. (13) GPP and FPP are key intermediate of essential oil biosynthesis which are driven by terpene synthases and other modifying enzymes (adapted and reconstructed from (Lv et al. 2024). Multiple arrows indicate: multiple steps; product color: orange, terpenoid; brown, essential oils; pink, phenolic compounds; blue, alkaloids.

Similarly, alkaloid synthesis follows the same regulatory mechanisms. For instance, tryptamine is synthesized in the shikimate pathway while secologanin is synthesized in MEP pathway. The levels of these compounds which influence catharanthine content in *Catharanthus roseus* are controlled by expression of genes like TDC (tryptophan decarboxylase), STR (strictosidine synthase) and SGD (strictosidine glucosidase) present upstream in the TIA biosynthetic pathway. Endophyte inoculation enhances the expression of gene PRX1 (peroxiredoxin1) significantly boosting the accumulation vindoline and catharanthine (Pandey et al., 2016a,b; Figure 4).

## 6 Regulatory cross-talk mechanisms

### 6.1 Signal crosstalk mediated by microbial elicitors

Microbes in the rhizosphere release certain chemical compounds known as elicitors that enable plants adapt to stress conditions by stimulating the production of secondary metabolites (Naik and Al-Khayri, 2016). These elicitors commonly include lipopolysaccharides (LPS), proteins or peptides such as flg22, 13-pep, chitin, β-glucans, and their fragments. Binding of elicitors to the receptors is the first step in plant microbe interaction. Plant cells contain pattern recognition receptors (PRRs) such as Leucine-rich repeat receptor like kinases (LRR-RLKs) which detect microbe associated molecular patterns (MAMPs) such as flagellin, chitin, β-glucans, etc. This recognition initiates PAMP-triggered immunity (PTI). The Transcription factor MYC2 plays a central role in Induced systemic resistance (ISR), regulated by the gene Non Expressor of Pathogenesis related Genes1 (NPR1). The phytohormones JA and ET are essential for ISR activation and their increased levels stimulate ROS production ultimately leading to secondary metabolite synthesis (Meena et al., 2020).

Elicitors may act by binding directly to the receptor or even without binding they initiate intracellular signaling cascades through the release of secondary endogenous messengers such as calcium ions (Ca^2+^) along with the production of reactive oxygen species (ROS), plant defense hormones and mitogen-activated protein kinases (MAPKs).

These secondary messengers enhance the expression of genes involved in the mevalonate (MVA) pathway, methylerythritol phosphate (MEP) pathway and phenylpropanoid pathway resulting in the accumulation of secondary metabolites. Microbes thus act as bio stimulants for various biosynthetic pathways through augmenting gene expression. Signal crosstalk among elicitors, secondary messengers, key metabolite genes and enzyme activation forms the basis of plant secondary metabolite biosynthesis mediated by microbial consortia.

### 6.2 Signaling molecules of elicitor induced pathways

Calcium signaling is closely associated with other pathways involved in secondary metabolite synthesis. Ca^2+^ channels located in the plasma membrane open in response to stimuli, causing an increase in cytosolic Ca^2+^ levels. Calcium-binding proteins such as Calmodulin and Calcium-dependent protein kinase (CDPK) sense and bind Ca^2+^, subsequently initiating downstream signaling cascades.

In response to microbial elicitors, NADPH oxidase enzymes which are commonly referred as Respiratory Burst Oxidase Homologs (RBOHs) produce ROS. These enzymes are embedded in the plasma membrane and possess a hydrophilic N-terminal region with two EF-hand motifs, indicating their dependency on Ca^2+^ for activity (Kurusu et al., 2015). CDPKs activate StRbohB triggering an oxidative burst that generates H_2_O_2_, ${\text{O}}_{2}^{-}$, OH^−^ and singlet oxygen. However, intracellular Ca^2+^ spikes are essential for ROS generation and RBOHs together with MAPK cascade, transmit signals from cell to cell amplifying ROS production. Besides Ca2+, signaling molecules such as SA, ET and JA have also been reported to induce ROS generation (Long et al., 2019).

Mitogen-Activated Protein Kinases (MAPKs) regulate downstream transcription factors through reversible phosphorylation and dephosphorylation (Ronkina and Gaestel, 2022). GTP-binding proteins (G-proteins) play a pivotal role in initiating the MAPK cascade. Upon elicitor perception, the cascade is triggered and the GTP bound proteins stimulates phospholipases C (PLC) resulting in formation of inositol triphosphate (IP3) and diacylglycerol (DAG). Ca^2+^ together with DAG activate Protein Kinase C (PKC) which phosphorylates MAPKs thereby driving transcription factors associated with secondary metabolism. PLC activation elevates cytosolic Ca^2+^ level which in turn activates CDPKs. In addition, CDPKs enhance the activity of NADPH oxidase which positively regulates PAL and HMGR gene expression, through ROS generation (Khan et al., 2022).

### 6.3 Crosstalk in phytohormonal signaling

Phytohormones such as auxin, gibberellin, ethylene, cytokinin, abscisic acid, salicylic acid and jasmonic acid play crucial roles in regulating the accumulation of secondary metabolites by interacting with one another either synergistically or antagonistically through complex signal transduction mechanisms and regulatory networks.

**Figure 5:**
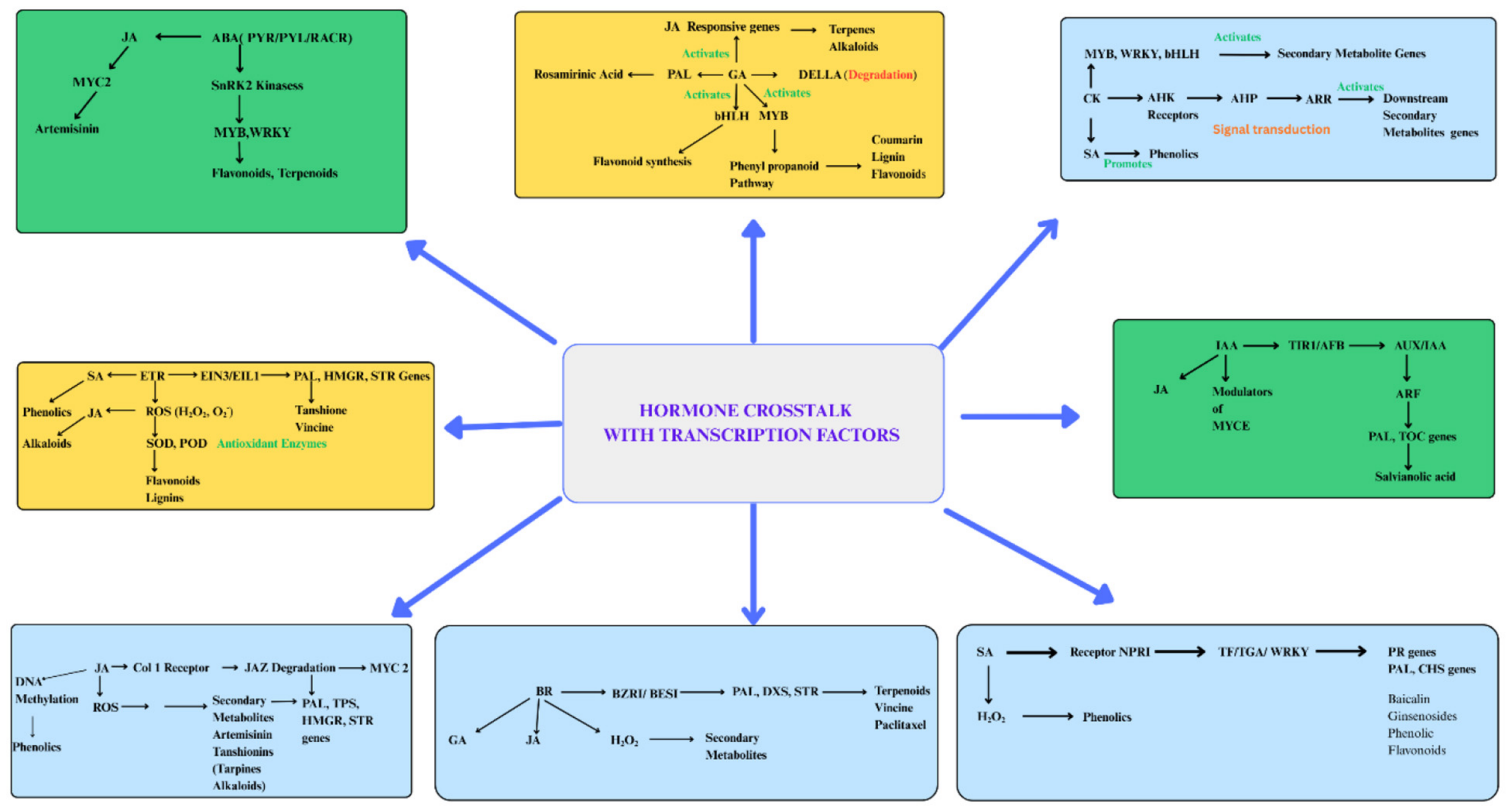
The figure depicts the intricate crosstalk between plant hormones and transcription factors that regulate secondary metabolite synthesis highlighting the integration of hormone signaling and transcriptional reprogramming for coordinated biosynthesis of terpenoids, phenolics, and alkaloids, etc.

In many plants, jasmonic acid (JA) which acts as mostly signaling molecule has been reported to activate numerous transcription factors that enhance the expression of biosynthetic genes. The relationship between JA and salicylic acid (SA) is predominantly antagonistic. SA binds to NPR1, which interferes with JA signaling at multiple levels, thereby modulating transcription factor activity. Additionally, SA increases the level of H_2_O_2_ in cell which acts as signaling molecules to activate the PAL, a key gene in Rosamirinic acid biosynthesis (Figure 5).

JA signaling involves JAZ (Jasmonate Zim Domain) proteins, which act as repressors by binding to transcription factors such as MYC2. JA perception occurs through the SCF-COI1-JAZ receptor complex, which mediates JAZ degradation. This degradation releases MYC2, enabling it to activate downstream genes that drive metabolite biosynthesis pathways (Chung et al., 2008).

On the other hand, Ethylene (ET) directly stimulates the expression of key biosynthetic enzyme genes via EIN3/EIL1 transcription factors. ET also interacts with other hormones such as JA and SA, as well as reactive oxygen species (ROS) to promote the biosynthesis of alkaloids and flavonoids.

Cytokinin activates genes by upregulating transcription factors such as MYB, WRKY, and bHLH. It acts synergistically with salicylic acid (SA**)** to promote phenolic accumulation, while in combination with jasmonic acid (JA) it can reduce the synthesis of certain metabolites within cells. Key enzymes of the phenylpropanoid pathway, including PAL, C4H and 4CL, are regulated by transcription factors such as TGA, which are activated by SA. Interestingly, SA can also interact synergistically with methyl jasmonate, enhancing the metabolite production.

Gibberellins promote the degradation of DELLA proteins which act as repressors of their activity. This degradation initiates downstream signaling cascades that activate the transcription factors controlling genes such as SmKSL1, PAL (Moreira et al., 2022).

Brassinosteroids (BRs) activate transcription factors BZR1/BES1, which upregulate key genes such as PAL, DXS and STR, thereby promoting metabolite accumulation. Through interaction with other plant hormones, BRs can also stimulate the polyamine biosynthesis pathway, enhancing the production of arginine-derived metabolites (Li et al., 2025).

Auxin regulates gene expression by releasing ARF (Auxin Response Factor) through the degradation of AUX/IAA suppressor proteins. At high auxin concentrations, auxin binds to the SCF-TIR1 ubiquitin ligase complex. This interaction promotes the ubiquitination and subsequent degradation of AUX/IAA proteins, thereby activating ARFs. Activated ARFs bind to Auxin Response Elements (AREs) in target gene promoters, initiating transcriptional programs of key genes PAL and SmKSL1 (Zhang et al., 2025).

Abscisic acid (ABA) signaling involves activation of Snf1-related protein kinases (SnRK2s) through phosphorylation. These kinases subsequently activate downstream transcription factors such as ABFs (ABA-responsive element-binding factors) and ABI5. Once activated, these transcription factors regulate the expression of genes such as CHS and IFS. This pathway enhances the production of flavonoids and other stress-related metabolites, linking ABA signaling to adaptive responses and metabolic regulation (Chang et al., 2022).

## 7 Transcriptional regulators and genes of secondary metabolism

Transcription factors (TFs) are DNA-binding proteins that regulate gene expression by interacting with promoter regions and facilitating initiation of transcription through RNA polymerase. By controlling the expression of key biosynthetic genes, transcription factors play a crucial role in coordinating and fine-tuning secondary metabolism. This section elaborates on the major TFs associated with genes of secondary metabolite pathways and highlights their regulatory cross-talk in shaping biosynthetic processes (Yang et al., 2012; Table 3).

**Table 3:** Transcription factors and key genes in plant secondary metabolite pathways.

Plants	Transcription factors	Genes regulated	Secondary metabolites	References
WRKY				
*V. vinifera*	VviWRKY24	VviSTS29	Resveratrol	(Vannier et al., 2019)
Wheat	TaWRKY70	ACT, DGK, GL1	Hydroxycinnamic acid amide	(Kajla et al., 2023)
*S. tuberosum*	StWRKY8	TYDC, NCS, COR2	Benzyl isoquinoline	(Yogendra et al., 2017)
*T. chinenesis*	TcWRKY1	DBAT	Taxol	(Cruz-Magalhães et al., 2022)
*C. roseus*	CrWRKY1	TDC, STR, D4H, and DAT	Indole alkaloid	(Liu et al., 2021)
*Artemisia annua*	AaWRKY6	DBR2	Artemisinin	(Wang et al., 2023)
MYB				
*A. thaliana*	AtMYB34, AtMYB51, AtMYB122	CYP79B2, CYP79B3, CYP83B1	Flavonoids	(Dubos et al., 2010)
*C. cinensis*	CsMYBF1	CHS	Flavonoids	(Liu et al., 2016)
*O. sativa*	MYB30, MYB55, MYB110	HCT, 4CL3, C4H, and PAL	Hydroxycinnamic acid amides	(Rabeh et al., 2025)
*D. gregaria*	PtMYB115	ANR1, LAR3	Proanthocyanin	(Wang et al., 2017)
*N. tabacum*	SbMYB8	CHS	Flavonoid	(Wang et al., 2024)
bHLH				
*C. roseus*	CrMYC2	ORCA-3	Alkaloid	(Jan et al., 2021)
*A. thaliana*	MdMYC2	DFR, F3H, uF3GT, and CHS	Sesquiterpenes	(Bao et al., 2020)
*M. truncatula*	TSAR1, TSAR2	HMGR	Saponin	(Mertens et al., 2016)
*O. sativa*	DPF (OsbHLH025)	OsCPS2, CYP99A2	Diterpenoid	(Zhou et al., 2024)
NAC				
*P. trifoliata*	PtrNAC72	ADC	Putrescine	(Wu et al., 2016)
*N. benthamiana*	ANAC042	CYP71A12, PAL1, and CYP71B15	Camalexin,	(Monsalvo et al., 2025)
*A. thaliana*	ANAC032	DFR, LDOX, and ANS,	Anthocyanin	(Mahmood et al., 2016)
*M. falcata*	MfNAC	GLO1	Glutathione	(Duan et al., 2017)
Norway spruce	PaNAC03	CHS, F3'H, and LAR3	Flavonoids	(Dalman et al., 2017)
bZIP				
*S. miltiorrhiza*	SmbZIP20, SmbZIP7, and AabZIP1	SmHMGR2, SmCPS1, SmKSL1, and SmCYP76AH1	Tanshione	(Zhang et al., 2018)
*Malus domestica*	MdHY5,	MdMYB10,	Anthocyanin	(An et al., 2017)
*Solanum lycopersicum*	SlHY5	QH6	Monoterpene	(Zhou et al., 2015)
*O. sativa*	OsTGAP1	OsKSL4 and OsCPS4	Terpenoid	(Yoshida et al., 2017)
AP2/ERF				
*Artemisia annua*	AaERF1 AaERF2	CYP71AV ADS	Artemisinin	(Yu et al., 2012)
*N. tabacum*	NtORC1/ERF221 NtJAP1/ERF10	CrPRX1	Vinblastine	(De Sutter et al., 2005)
*S. lycopersicum*	GAME9	HMGR, GAMEs, C5-SD, CAS, and SGTs,	Steroidal glycoalkaloids	(Thagun et al., 2016)
*O. sativa*	OsERF922	PR, PAL	Phytoalexin	(Mengchao et al., 2023)
*A. thaliana* and *T. aestivum*	TaERF13-2B	AtP5CS1, AtRD29A, AtRDREB2A, AtCOR15A, AtMYB15, AtERD10, AtLTI30, AtKIN1, AtGSTU19	Proline	(Yu et al., 2025)

### 7.1 WRKY transcription factors (TFs)

WRKY gene expression is primarily associated with the regulation of defense-related secondary metabolite biosynthesis and they are among the most extensively studied TFs (Chen et al., 2017). Members of this family possess a conserved domain of approximately 60 amino acids called as WRKY domain, which is responsible for gene regulation through interaction with W-box cis acting elements (TTGACT/C) as it shows higher affinity for DNA binding in the promoter regions of target genes.

WRKY TFs have several roles in plants processes such as development, seed dormancy and germination but prominently involved in stress responses. They are often activated by wounding or jasmonic acid signaling leading to the expression of various genes associated with the biosynthesis of alkaloids, terpenoids, flavonoids, phenols and related compounds (Phukan et al., 2016).

**Figure 6:**
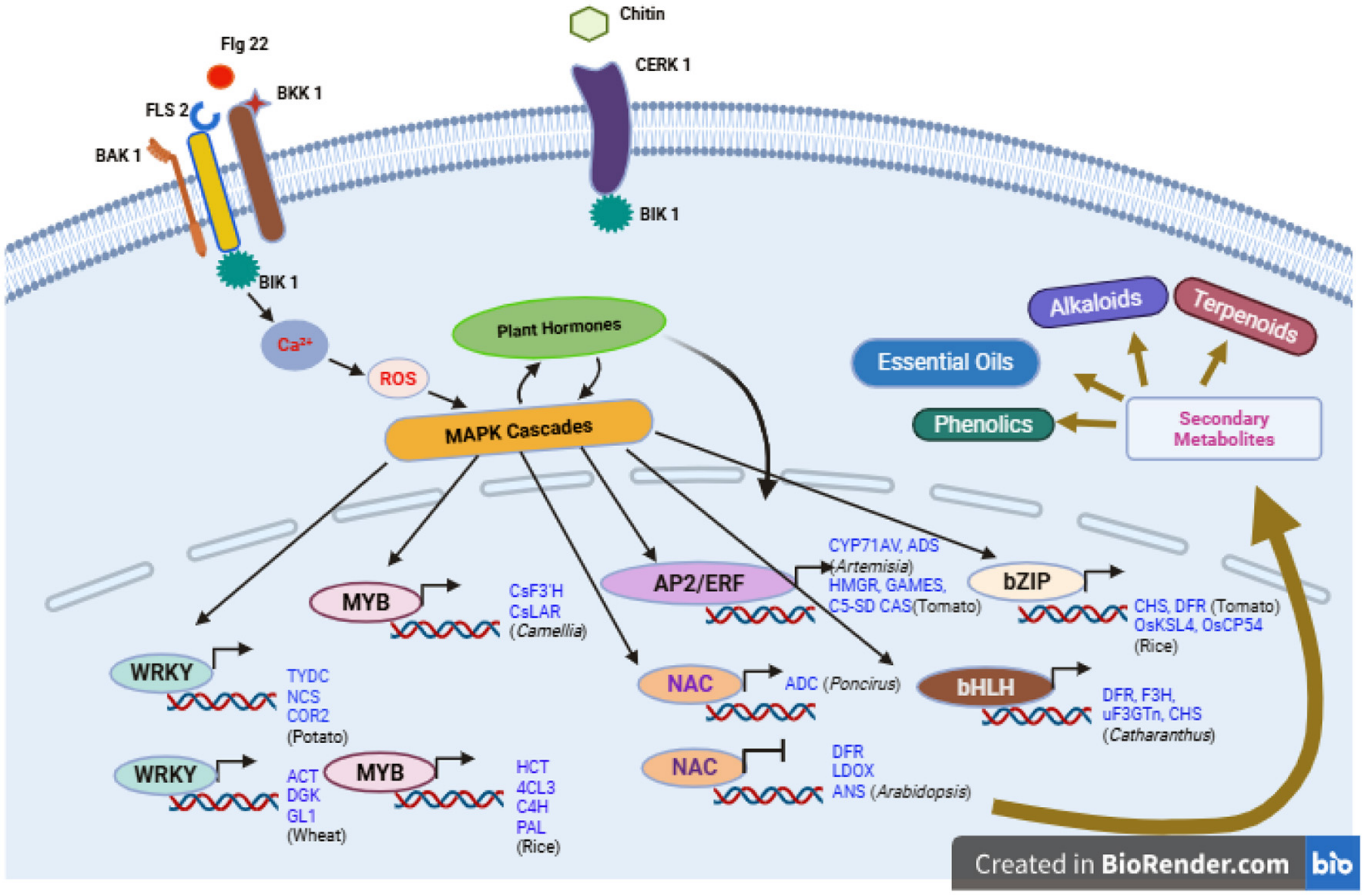
Microbial consortia modulating plant secondary metabolism in *Arabidopsis* as a model system. Binding of flg22 to the receptor FLS2 promotes its association with co-receptors BAK1 and BKK1, leading to their phosphorylation. This in turn induces phosphorylation of BIK1, which then dissociates from receptor complex. Released BIK1 results in calcium spike and subsequent ROS burst. The ROS burst activates the MAPK cascades ultimately stimulating the transcription factors that drive the expression of genes involved in secondary metabolite biosynthesis. In parallel, MAPK signaling also engages hormone pathways, which modulate transcription factors activity indirectly by promoting the degradation of repressor proteins bound to them. Similarly, chitin perception occurs through the CERK1 receptor which interacts with BIK1 but not with BAK1 triggering a downstream signaling cascade.

For instance, TaWRKY70 has been reported to regulate and modulate the phenylpropanoid pathway by inducing hydroxycinnamic acid amide (HCAA) related genes such as ACT, DGK and GL1 in wheat (Kage et al., 2017). Similarly, the transcription factor StWRKY8 enhances the expression of three genes TYDC, NCS and COR2 genes, involved in the biosynthesis of benzyl isoquinoline alkaloid in potato (Yogendra et al., 2017). Indole alkaloid accumulation occurs through the expression of the TDC gene, regulated by CrWRKY1 which itself is activated by jasmonic acid signaling. Additionally, in *Salvia sclarea*, SsWRKY18, SsWRKY40, and SsMYC2 collectively control biosynthesis of abietane type diterpene (Alfieri et al., 2018). Thus, multiple WRKY transcription factors are engaged in the transcriptional activation of secondary metabolite biosynthesis pathways (Kajla et al., 2023; Figure 6).

### 7.2 MYB transcription factors (TFs)

MYB Transcription factors (TFs) are involved not only in the biosynthesis of secondary metabolites but also in plant growth and reproduction. MYB proteins contain two distinct regions: a highly conserved N-terminal DNA-binding MYB domain and a variable C-terminal region. The DNA binding domain consists of 50–53 amino acid residues arranged in four imperfect repeats. Each repeat has three α-helices, out of which the second and third helices are responsible for gene recognition. Based on their DNA-binding domains, MYB TFs are classified into four subclasses: 1R, R2R3, 3R, and 4R (Dubos et al., 2010).

Among these, the R2R3 family plays a crucial role in regulating genes associated with secondary metabolite biosynthetic pathways. For instance, AtMYB113, AtMYB75, and AtMYB90 are R2R3 MYBs and they regulate anthocyanin accumulation in plants by modulating the phenylpropanoid pathway in *Arabidopsis thaliana*. Similarly, AtMYB29 and AtMYB76 regulate aliphatic glucosinolate accumulation, while AtMYB34, AtMYB51, and AtMYB122 control indole glucosinolate accumulation by regulating CYP79B2, CYP79B3, and CYP83B1 in the tryptophan biosynthetic pathway (Dubos et al., 2010).

In *Camellia sinensis*, transcription factors CsMYB2 and CsMYB26 bind to the promoters of CsF3'H and Cs LAR, leading to flavonoid accumulation (Nisha et al., 2018). In *Oryza sativa*, MYB30, MYB55, and MYB110 upregulate the expression of HCT, 4CL3, C4H and PAL, resulting in the accumulation of hydroxycinnamic acid amides (HCAAs) through modulation of the phenylpropanoid pathway (Kishi-Kaboshi et al., 2018).

### 7.3 bHLH transcription factors (TFs)

These group of transcription factors play a pivotal role in stress response mechanisms in plants. They are characterized by the presence of approximately 60 amino acids forming a bipartite conserved domain. This domain consists of two parts: a basic amino acid region and the helix-loop-helix (HLH) region. The N-terminal basic helix facilitates DNA binding, while the two α-helices located in the C-terminal end enable the formation of homo or heterodimeric complexes with MYB proteins and thereby controlling specific gene expression.

bHLH TFs modulate hormonal signaling, particularly jasmonic acid and are known to regulate the biosynthesis of anthocyanins, alkaloids, diterpenoids, phytoalexins, and saponins (Sun et al., 2018). In *Arabidopsis thaliana*, bHLH04, bHLH05, and bHLH06, in association with MYB51, upregulate the biosynthesis of glucosinolates (GLs), anthocyanin, and flavonoids via phenylpropanoid pathway (Frerigmann et al., 2014). CrMYC2, a bHLH family protein, acts as an activator of jasmonic acid–responsive ORCA3 gene expression, regulating alkaloid biosynthesis in *Catharanthus roseus* (Zhang et al., 2011). Similarly, MdMYC2 activates anthocyanin biosynthetic genes such as DFR, F3H, uF3GTn, and CHS. Through GA and JA signaling pathways and by interacting with DELLA proteins, MdMYC2 also promotes the synthesis of sesquiterpenes in *A. thaliana*.

TSAR1 and TSAR2 are bHLH TFs that activate HMGR genes, leading to saponin accumulation in *Medicago truncatula* (Mertens et al., 2016). In some cases, different TFs form complexes, such as the MYB-bHLH-WDR complex, which enhances anthocyanin and proanthocyanidin regulation in *A. thaliana* (Nemesio-Gorriz et al., 2017). Likewise, diterpenoid phytoalexin factors regulate the accumulation of diterpenoid phytoalexins in rice by controlling the expression of genes CPS2 and CYP99A2 (Yamamura et al., 2015).

### 7.4 NAC transcription factors (TFs)

NAC transcription factors (NAC TFs) represent a large family of proteins associated with the regulation of gene expression under both biotic and abiotic stress conditions. They are characterized by the presence of a NAC domain, which consists of a highly conserved N-terminal region functioning as a DNA-binding site and a highly diverse C-terminal region that acts as a transcriptional regulatory domain. Members of this protein family play crucial roles in modulating specific genes involved in the synthesis of secondary metabolites (SMs), particularly when plants encounter stress. For instance, ANAC042 in association with CYP71A12, CYP71A13, and CYP71B15 controls the biosynthetic genes of camalexin, a well-known phytoalexin. The accumulation of camalexin enhances plant resistance to *Alternaria brassicicola* infection (Saga et al., 2012). NAC TFs also contribute to the regulation of reactive oxygen species (ROS) homeostasis. For example, the ADC gene which is responsible for putrescine biosynthesis, plays a role in ROS regulation and is controlled by the transcription factor PtrNAC72 (Wu et al., 2016). Additionally, ANAC032 negatively regulates genes such as DFR, LDOX and ANS, which are associated with anthocyanin biosynthesis (Mahmood et al., 2016).

### 7.5 bZIP transcription factors (TFs)

bZIP transcription factors are identified by a conserved leucine zipper domain coupled with a positively charged DNA-binding site. They play crucial roles in diverse biological processes, particularly under osmotic, nutrient and oxidative stress conditions. For instance, bZIP20, SmbZIP7, and AabZIP1 have been shown to enhance the synthesis of secondary metabolites such as tanshione in *Salvia miltiorrhiza* and artemisinin in *Artemisia annua* (Zhang et al., 2018). Similarly, MdHY5, a light-responsive bZIP TF in apple, regulates anthocyanin accumulation either independently or in combination with MdMYB10 (An et al., 2017). In tomato, SlHY5 controls anthocyanin accumulation by regulating the expression of CHS and DFR genes and also modulates the expression of QH6, which is responsible for monoterpene biosynthesis (Zhou et al., 2015). In rice, OsTGAP1 regulates OsKSL4 and OsCPS4 genes thus enhancing the synthesis of terpenoid phytoalexins, while also modulating the MEP pathway (Yoshida et al., 2017).

### 7.6 AP2/ERF transcription factors

AP2/ERF transcription factors contain the AP2 DNA-binding domain, a conserved domain first reported in the floral homeotic gene APETALA2 (AP2) of *Arabidopsis thaliana*, from which the family derives its name. Based on variations in additional conserved domains, they are classified into four subfamilies: AP2, ERF, RAV, and DREB (Zhou et al., 2016).

Biosynthesis of terpenoid indole alkaloids (TIAs) is regulated by ORCA and ORCA2, members of the AP2/ERF family, through the enhancement of gene expression. ORCA3, a jasmonic acid–activated TF, induces the expression of genes encoding strictosidine synthase and tryptophan decarboxylase by binding specifically to the JERE element present in their promoter regions. In *Artemisia annua*, two jasmonic acid–responsive AP2/ERF TFs, AaERF1, and AaERF2, regulate CYP71AV and ADS, thereby enhancing the synthesis of artemisinin and artemisinic acid by binding to CBF2 and RAA sites (Yu and He, 2012).

**Figure 7:**
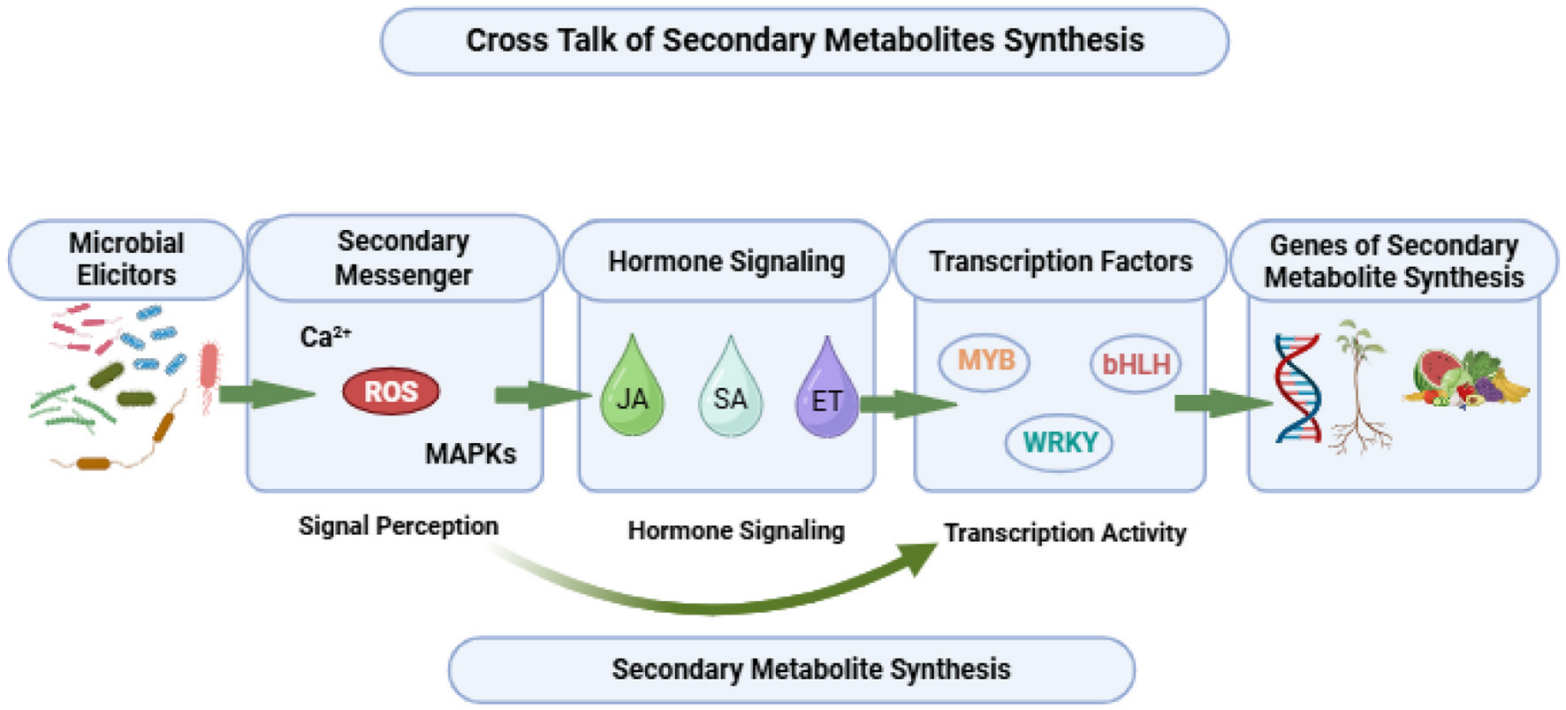
Microbial elicitors activate secondary messengers, which in turn trigger hormone signaling pathways and transcription factors to produce secondary metabolites through a complex cross-talk mechanism. While the integrative model provides a conceptual framework, future validation requires incorporation of quantitative network analysis approaches such as co-occurrence networks, correlation-based microbial interaction matrices, and dynamic modeling of signaling cascades. These approaches can help identify keystone taxa, quantify interaction strengths, and resolve unresolved aspects such as microbial antagonism and stability within consortia. Additionally, gap-filling hypotheses regarding functional redundancy and competitive exclusion within microbial communities needs to be experimentally validated to improve predictive modeling of consortium performance.

AP2/ERF TFs are also involved in the biosynthesis of nicotine alkaloids in tobacco. For instance, NtORC1/ERF221 and NtJAP1/ERF10 control the transcriptional regulation of the PMT gene (De Sutter et al., 2005). Additionally, they have been reported to regulate CrPRX1 in vinblastine biosynthesis. GAME9, another AP2/ERF family protein, promotes the biosynthesis of steroidal glycoalkaloids by regulating the expression of HMGR, GAMEs, C5-SD, CAS, and SGTs, which are key components of plant defense mechanisms (Thagun et al., 2016). Similarly, PnERF1 enhances saponin accumulation by upregulating the expression of HMGR, EPS, DS, and SS genes (Deng Bing et al., 2017; Figure 7).

## 8 Conclusion

This review highlights recent applications and future perspectives of microbial consortia with a special focus on their regulation mechanisms in secondary metabolite production. For specific crops and environmental conditions, however a tailored microbial consortium must be formulated for precision agriculture. Yet, the beneficial effects observed with a microbial model consortium are not always consistent when compared to those obtained from a single bacterial strain. For example, the individual application of two strains of *Bacillus velezensis* BMH and INV showed greater suppression of galls and eggs of *Meloidogyne incognita* in tomato roots compared to their combined application (Cruz-Magalhães et al., 2022). These instances indicate the existence of potentially complex interactions among microorganisms in a consortium. Thus, understanding the various interactions among heterogenous species of microorganisms, as well as compatibility among themselves with different plant genotypes is crucial (Mishra et al., 2022). Several studies have highlighted concerns regarding certain bacteria such as *Pseudomonas* and *Burkholderia* species. While these organisms demonstrate plant growth promoting properties, at the same time they are also pathogenic to humans. Therefore, before being incorporated in to commercial microbial consortium, their potential risks must be thoroughly evaluated and addressed.

PGPMs promote plant growth and development through mutualistic interactions or by protecting the plants from pathogenic infections. They enhance secondary metabolites biosynthesis in several ways, including activation of host plant's immune system. However, the developmental stage of plants is a crucial factor affecting the process. In a study, examining the correlation between secondary metabolite content and microbial diversity, dynamic correlations were observed between microorganisms inhabiting roots and root metabolism indicating their influence on developmental stages (Csorba et al., 2022).

Microbial elicitors like proteins, oligosaccharides and lipids play an important role in inducing plant defense responses thereby stimulating the accumulation of bioactive compounds. Synthesis of these compounds can also occur through the release of volatile chemicals a method that does not require physical contact with plant.

Microbes mediated elicitation plays a pivotal role as a dynamic regulatory strategy through which plants reprogram their metabolism to enhance the synthesis of secondary metabolites. Beneficial microbes of a consortium release elicitors, such as microbe-associated molecular patterns (MAMPs) which bind to specific receptors on the plant cell membrane. This binding initiates a downstream signaling cascade involving molecules such as reactive oxygen species (ROS) nitric oxide (NO) and calcium fluxes, etc. Hormonal cross talk particularly among jasmonic acid, salicylic acid, ethylene and auxin further amplifies these signals, coordinating growth regulation and defense responses. These signaling events subsequently activate transcription factors such as MYB, WRKY, AP2/ERF, bHLH, NAC, etc. which function as molecular switches by turning on or off genes encoding key enzymes in secondary metabolic pathways.

The regulatory system of metabolite biosynthesis arising from interactions among elicitors, signaling molecules, plant hormones, transcription factors and key genes is highly modular and sophisticated. Microbial elicitation activates transcription factors through phosphorylation cascades, epigenetic modification and feedback loops. This integrated network exerts precise control over biosynthetic gene clusters resulting in elevated accumulation of alkaloids, flavonoids, terpenoids, essential oil and phenolic compounds. Thus, microbial consortia amplify the secondary metabolite production by providing synergistic elicitors and maintaining a healthy rhizosphere environment which represents an eco-friendly and sustainable strategy aligning with global efforts to reduce chemical inputs and restore degraded ecosystems.

Many of these plant secondary metabolite have therapeutic potential acting as a boon to humans. However, it is evident that not all bioactive compounds produced by microorganisms are promotive in nature; some may also produce regulatory substances. Most studies provide evidence on the elicitation mechanisms but the pathways inhibiting the synthesis of SMs cannot be ruled out. To explore how plants employ and stimulate beneficial microorganisms, it is essential to identify root exudates, signaling molecules and key players in the rhizosphere microbiome. It needs a detailed understanding on the molecular and biochemical pathways that can harness the benefits of soil microbiomes and their host crops. Although numerous research publications have addressed these issues, field studies still remain limited. Therefore, developing a model consortium using beneficial microorganisms in a mixed form holds great promise for enhancing crop yield and quality.

## 9 Future prospects

Future studies should include unique microbial resources from different ecological niches or habitats, exploring their intricate interactions in depth and their ability to regulate plant secondary metabolism. Through real time quantitative PCR and diagnostic probes, such as metagenomics, meta-transcriptomics and targeted DNA sequencing, mechanisms of plant-microbe interactions can be studied by monitoring the establishment and stability of specific microbes in the field. These approaches can help develop a more general model of microbial consortia for specific crops or for customize applications providing reliable and eco-friendly solutions to the challenges in modern agricultural systems.

Nevertheless, the resilience and reliability of microbial consortia under dynamic conditions remain insufficiently characterized, and the translation of laboratory findings to field-level applications continues to pose significant challenges. The complex interactions among the microbes within a consortium including the ways they signal each other to coordinate the secondary metabolism, necessitate advanced tools such as monitoring, modeling and engineering. The integration of computational biology, synthetic biology and systems biology is crucial for unraveling these complexities, thereby facilitating the design of microbial consortia with enhanced and optimized functionality.

Future research should increasingly integrate multi-omics approaches, including metagenomics, metatranscriptomics, and metabolomics, to achieve a systems-level understanding of microbial consortia. CRISPR-Cas technology has revolutionized the design of microbial consortium in producing plant secondary metabolites. By integrating multi omics, systems biology and bioprocess engineering strategies this approach will enable more robust consortium production. Beyond enhancing the productivity, it will streamline metabolic reprogramming and strengthen environmentally responsive traits within microbial communities. These advances represent a paradigm shift in microbial biotechnology.

Synthetic biology approaches offer promising opportunities for designing engineered microbial consortia with enhanced functional stability and precision. Integration of artificial intelligence and machine learning can further enable predictive modeling of microbial interactions and optimization of consortia performance under diverse environmental conditions.
